# Compound and heterogeneous relationships between climate extremes and global net migration

**DOI:** 10.1038/s41558-026-02752-4

**Published:** 2026-09-11

**Authors:** Kristina Petrova, Karim Zantout, Sandra Zimmermann, Venla Niva, Matti Kummu, Katja Frieler, Jacob Schewe

**Affiliations:** 1 Potsdam Institute for Climate Impact Research (PIK), Member of the Leibniz Association, Potsdam, Germany; 2 University of Mannheim, Mannheim, Germany; 3 Karlsruhe University of Applied Sciences, Karlsruhe, Germany; 4 Aalto University, Espoo, Finland; 5 Water and Development Research Group, Aalto University, Espoo, Finland

**Keywords:** Governance, Climate change

## Abstract

Climate extremes are increasingly disrupting livelihoods and shaping patterns of human mobility. Their longer-term and compounding relationships with migration remain understudied, particularly at the subnational scale. In this study, by analysing global subnational net migration data, we show that associations between climate extremes and migration vary by hazard type, timing, consecutive events, income level and pre-existing migration context. Floods are associated with decreased net migration across contexts, with effects lasting several years and appearing stronger in lower-income areas. Droughts, by contrast, show associations with net migration primarily in higher-income settings, with effects generally short-term. Tropical cyclones correspond to decreased net migration primarily in lower-income areas, with effects lasting over several years. For compound exposure, droughts show the clearest patterns, with prolonged exposure linked to reduced migration inflows in population-attracting areas. These results highlight how climate extremes interact with local socioeconomic contexts to shape mobility.

## Abstract (web summary)

Climate extremes influence migration, but their long-term and compounding impacts are understudied. The authors show that associations between climate extremes and migration vary by hazard type, timing, the occurrence of consecutive events and local socioeconomic conditions.

## Main

Climate change increases the frequency and severity of extreme weather events such as storms and floods and contributes to slower, but equally damaging processes such as drought, desertification and sea-level rise^1^. These changes threaten ecosystems and human populations, which demands urgent action to mitigate and adapt. In response to such stresses, households use a range of coping mechanisms, from adopting new technologies and using savings to relying on community support networks^2–4^. When these strategies are insufficient, relocation can serve as an adaptation measure to protect livelihoods and diversify income sources.

Disasters linked to natural hazards have displaced roughly 265 million people over the past decade^5^. Research on climate and mobility has examined how short-term weather variability^6,7^, floods^8–10^, droughts^11,12^, storms^13,14^ and wildfires^15,16^, shape patterns of human migration, although most studies examine only specific countries or regions or focus on single hazards^17^.

Environmental stress can increase migration aspirations, particularly towards domestic destinations^18^. Rapid-onset events, such as floods and cyclones, tend to produce short-term movements, whereas slow-onset events, such as droughts and crop failures, tend to generate more gradual responses^19,20^. The distinction reflects differences in the speed with which impacts unfold and the recovery time. Sudden shocks cause abrupt losses that can cause temporary relocation^21,22^, whereas slower hazards unfold over time, allowing for in-place adaptation^23^. These patterns are not universal. For instance, ref. ^24^ find that rapid-onset events may also inhibit migration. Single events, however, represent only part of the picture. When extreme events occur repeatedly, their impacts can accumulate, gradually eroding coping capacity and reducing the ability of households to use typical adaptation strategies^25–27^. Taking into account such prior exposure is, therefore, important for understanding migration responses.

Using harmonized climate and subnational migration data, we extend this literature by analysing how droughts, floods, tropical cyclones and crop failures are associated with patterns of human mobility. These hazards represent those for which globally consistent, high-resolution data are currently available. Our contributions are threefold. First, we examine four distinct hazard types over two decades, capturing both immediate and delayed migration responses. Second, we assess how these associations differ by pre-existing migration context and income levels. Finally, we analyse the relationship between consecutive events and cumulative changes in migration, offering a global perspective on the dynamic links between climate stress and human mobility.

## Research design

**Fig. 1:**
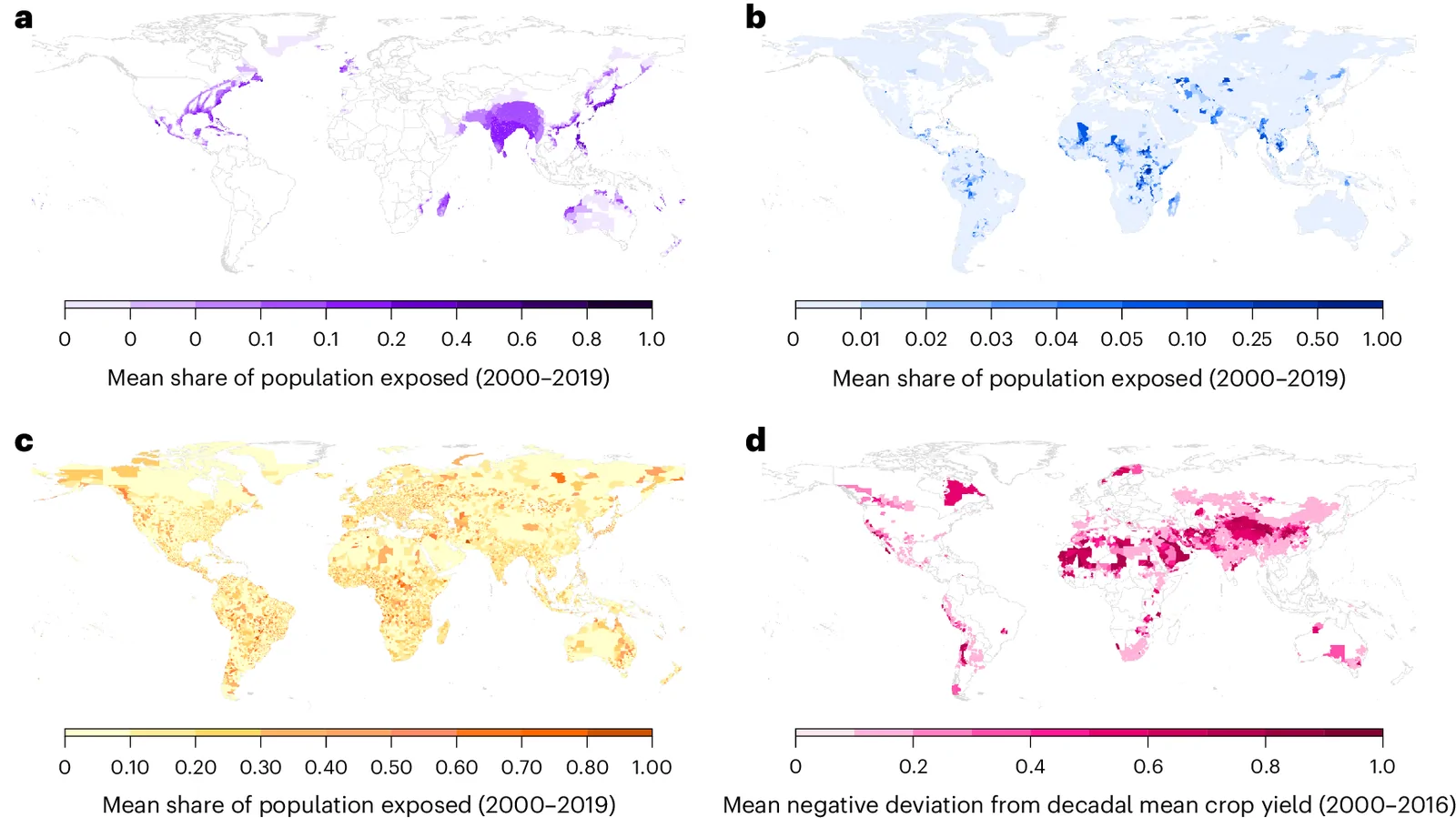
Spatial distribution of climate shocks between 2000 and 2019. **a**–**d**, Areas affected by tropical cyclones (**a**), floods (**b**), droughts (**c**) and crop failure (available through 2016) (**d**). Darker shades indicate stronger or more frequent impacts. All variables are aggregated at Admin-2. Basemaps in **a**–**d** from GADM v.4.1 (https://gadm.org/).

To study the relationship between climate hazards and net migration, we use harmonized data from the Inter-Sectoral Impact Model Intercomparison Project (ISIMIP) project^28^ and subnational net migration estimates from ref. ^29^. We show regional differences in cumulative exposure to tropical cyclones (Fig. 1a), floods (Fig. 1b), droughts (Fig. 1c) and crop failures (Fig. 1d) over 2000–2019 (with crop failure data available until 2016). Darker shades represent areas more strongly impacted over the years. All hazard indicators are constructed under a unified intensity–exposure framework to ensure cross-hazard comparability, with each hazard defined as the share of the local population exposed above hazard-specific thresholds.

For the outcome of interest, we use annual estimates of net migration at the second administrative level (Admin-2) for 216 countries and territories from 2000 to 2019^29^. Net migration represents the balance between in- and out-migration and is derived from total and natural population change. Although the data enable fine-grained spatial analysis, uncertainty remains higher in regions with sparse or low-quality census data. To improve comparability across areas and reduce the influence of outliers, we use the logarithm of the absolute value of net migration rates per 1,000 people.

Recognizing that migration is shaped by several contextual factors beyond climate, our models incorporate measures of political, security and socioeconomic conditions, including conflict intensity^30,31^, ethnic group representation in political governance^32^, regime type^33^, economic activity and human development^34,35^, population size^36^ and administrative area size. Countries are grouped by World Bank income classification.

Detailed information on data sources, data construction and validation and indicator definitions are provided in the Methods.

### Associations between single climate event and net migration

**Fig. 2:**
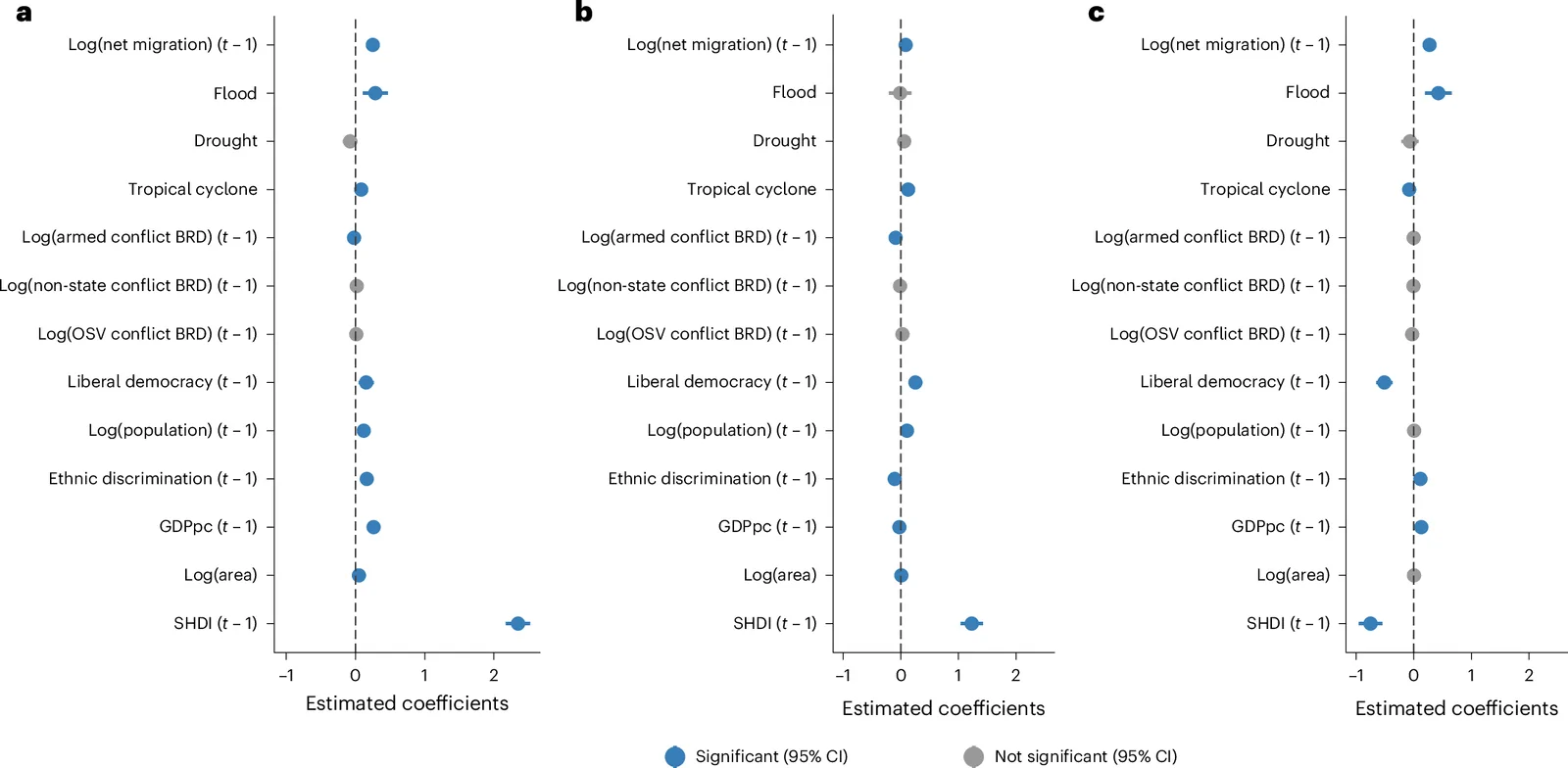
Global associations between single climate events and net migration. **a**–**c**, The associations between climate extremes and net migration for the full sample (*n* = 528,459) (**a**), negative net migration (*n* = 189,944) (**b**) and positive net migration (*n* = 111,524) (**c**). Points show estimated coefficients from mixed-effects linear regressions with random intercepts for year and country and error bars represent 95% confidence intervals (CIs) around the estimated means. Statistically insignificant coefficients are greyed out. BRD, battle-related deaths; GDPpc, gross domestic product per capita; OSV, one-sided violence; SHDI, Subnational Human Development Index; *t*, year. Source data

We first estimate mixed-effects linear regressions for three groups: the full sample (Fig. 2a), districts with over 14 years of negative average net migration (more people leaving; Fig. 2b) and districts with over 14 years of positive net migration (more people arriving; Fig. 2c). Districts are classified by the direction of net migration, positive or negative, for at least 14 of 20 years, distinguishing consistently out- and in-migrating areas from those with more variable patterns. Details on the model specification and robustness checks are provided in the Methods.

Floods in the same year are associated with higher net migration, suggesting increased in-migration or reduced out-migration. Given that annual net migration estimates rely on the interpolated gridded population data of WorldPop rather than observed populations, this same-year association should be interpreted with caution. The annual resolution of the migration data also means that migration in year *t* may partly precede flood events in the same year, potentially weakening the observed association. Nevertheless, we report same-year effects because short-lived responses may not be detectable at longer lags. The association appears in both the full sample and in positive net-migration areas. Tropical cyclones are also generally associated with increases in positive net migration in the same year, meaning fewer people leave and, in some cases, more move in. In areas that typically attract migrants, cyclones are associated with reduced in-migration, indicating temporary decreases in the attractiveness of such destinations. For droughts, we find no statistically significant association with net migration in the contemporaneous period. This lack of an immediate response is consistent with the slow-onset nature of droughts, whose impacts on livelihoods and migration incentives typically unfold over longer horizons rather than triggering abrupt population movements.

**Extended Data Fig. 1:**
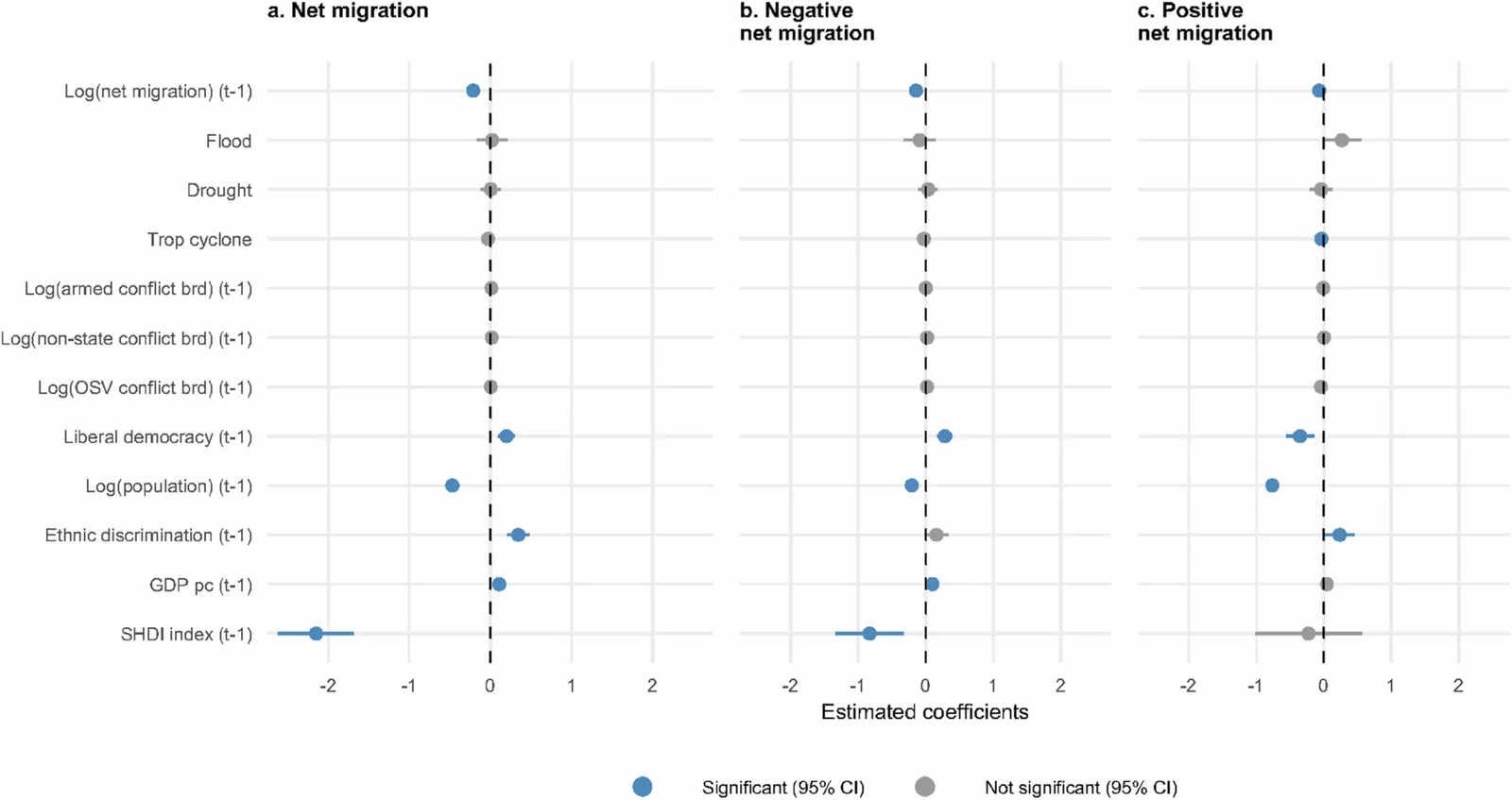
Fixed-effects regression estimates of the association between climate hazards and net migration. Panels present the associations between single climate extremes and net migration for the full sample (**a**; n = 536,795), negative net-migration districts (**b**; n = 191,236), and positive net-migration districts (c; n = 115,951). Results show estimated coefficients from fixed-effects linear regressions with year and second-order administrative unit (Admin-2) fixed effects; error bars represent 95% confidence intervals. Statistically insignificant coefficients are greyed out. Corresponding regression coefficients are reported in Extended Data Table 1. Source data

In additional analyses, crop failure shows no significant association across any samples (Supplementary Fig. 8 and Supplementary Table 4). Robustness tests with country- and year-fixed effects (Supplementary Fig. 7 and Supplementary Table 3) yield similar patterns, while models with region- and year-fixed effects (Extended Data Fig. 1 and Extended Data Table 1) indicate that floods have a positive association with net migration in net-positive areas only at the 10% confidence level. By contrast, tropical cyclones remain negatively associated with net migration in areas that usually attract people, consistent with a temporary reduction in in-migration following such events. To assess model performance, we compared specifications using the Akaike information criterion (AIC) and out-of-sample cross-validation (Supplementary Fig. 9). Both measures show that adding climate hazards alongside sociopolitical variables improves model fit, confirming that climate extremes contribute explanatory power beyond socioeconomic context. Further details on robustness checks and model comparisons are provided in the Methods.

Examining the covariates, higher economic activity, human development, stronger democratic institutions and larger population or area are all associated with higher net-migration rates, consistent with prior findings^29^. Conversely, greater conflict intensity corresponds to lower net migration, possibly reflecting restricted mobility or displacement within affected areas.

### Immediate and delayed effects on net migration

We extend our analysis to examine both immediate and delayed effects of climate extremes on net migration using a hierarchical Bayesian panel vector autoregressive (BPVAR) model. This framework captures dynamic interdependencies between climate hazards and migration over several years within each district, while estimating average effects across districts through hierarchical modelling that accounts for both shared patterns and local variation. This approach allows us to explore how migration patterns evolve following extreme events across diverse geographic and socioeconomic contexts. Full model details, estimation procedure and diagnostics are described in Methods.

**Fig. 3:**
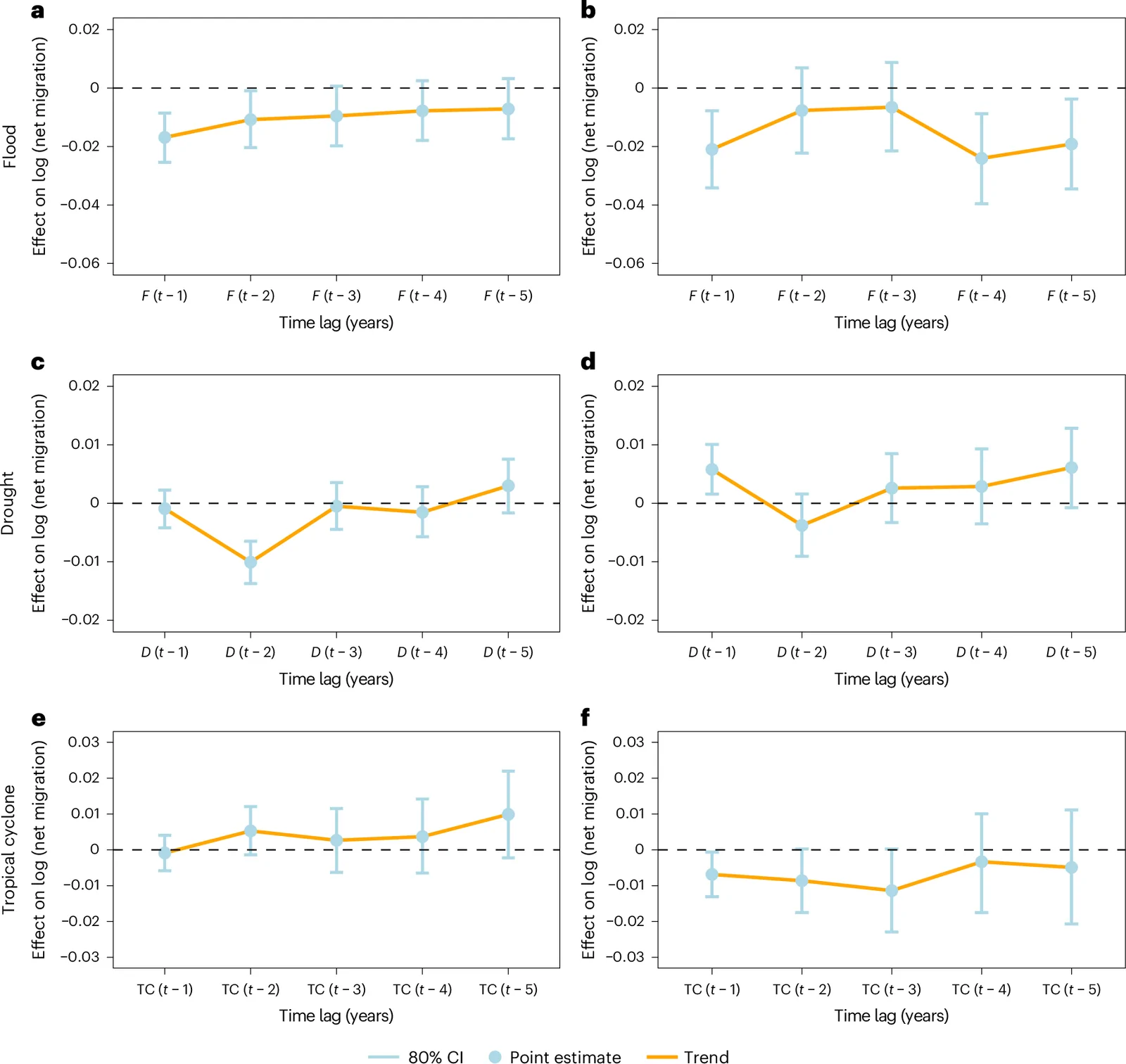
Climate events over a 5-year time lag and net migration. Associations between climate extremes and net migration estimated using a hierarchical BPVAR model (250,000 burn-in iterations, 250,000 post-burn-in iterations with thinning of 20). The unit of study is the district–year observation; results are based on districts with negative net migration (*n* = 189,944 district–year observations; left) and positive net migration (*n* = 111,524 district–year observations; right). Points show posterior mean coefficients and error bars represent 80% CIs. **a**–**f**, Results are shown separately for negative net migration (**a**,**c**,**e**) and positive net migration (**b**,**d**,**f**) across floods (*F*; **a**,**b**), droughts (*D*; **c**,**d**) and tropical cyclones (TC; **e**,**f**). Source data

**Extended Data Fig. 2:**
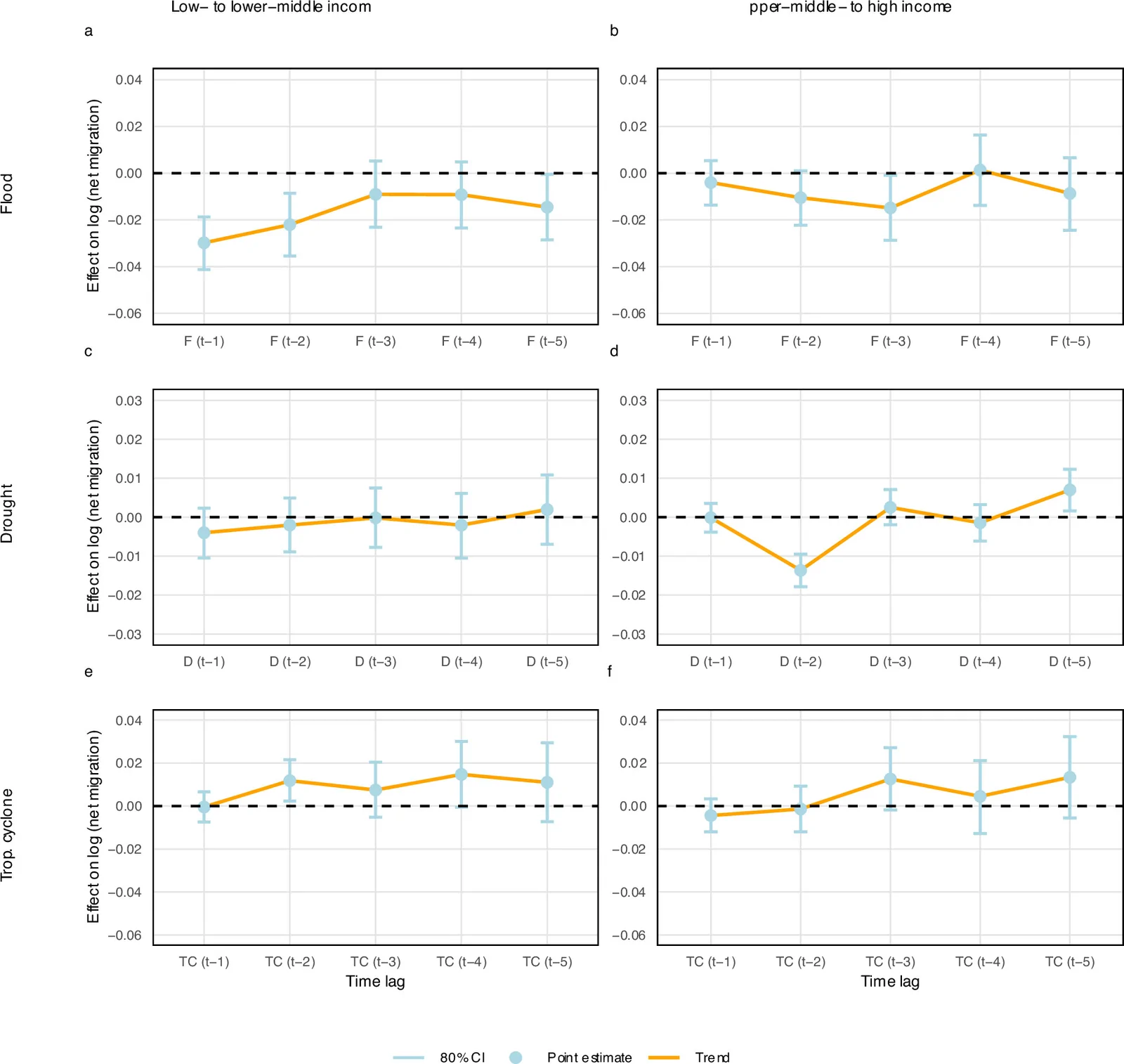
Associations between single climate hazards and net migration over a five-year lag in the negative net-migration sample, split by income group. Climate events (over a 5-year time lag) and net migration. Results show the associations between climate extremes and net migration in the negative net-migration sample, split by income groups: **a**, floods, low- to lower-middle-income countries (n = 65,819 district-year observations); **b**, floods, upper-middle- to high-income countries (n = 29,518 district-year observations); **c**, droughts, low- to lower-middle-income countries (n = 89,603 district-year observations); **d**, droughts, upper-middle- to high-income countries (n = 127,508 district-year observations); **e**, tropical cyclones, low- to lower-middle-income countries (n = 22,710 district-year observations); **f**, tropical cyclones, upper-middle- to high-income countries (n = 17,757 district-year observations). Coefficients are estimated using a hierarchical Bayesian panel vector autoregressive model (250,000 burn-in iterations, 250,000 post-burn-in iterations with thinning of 20), and error bars represent 80% credible intervals. Source data

**Extended Data Fig. 3:**
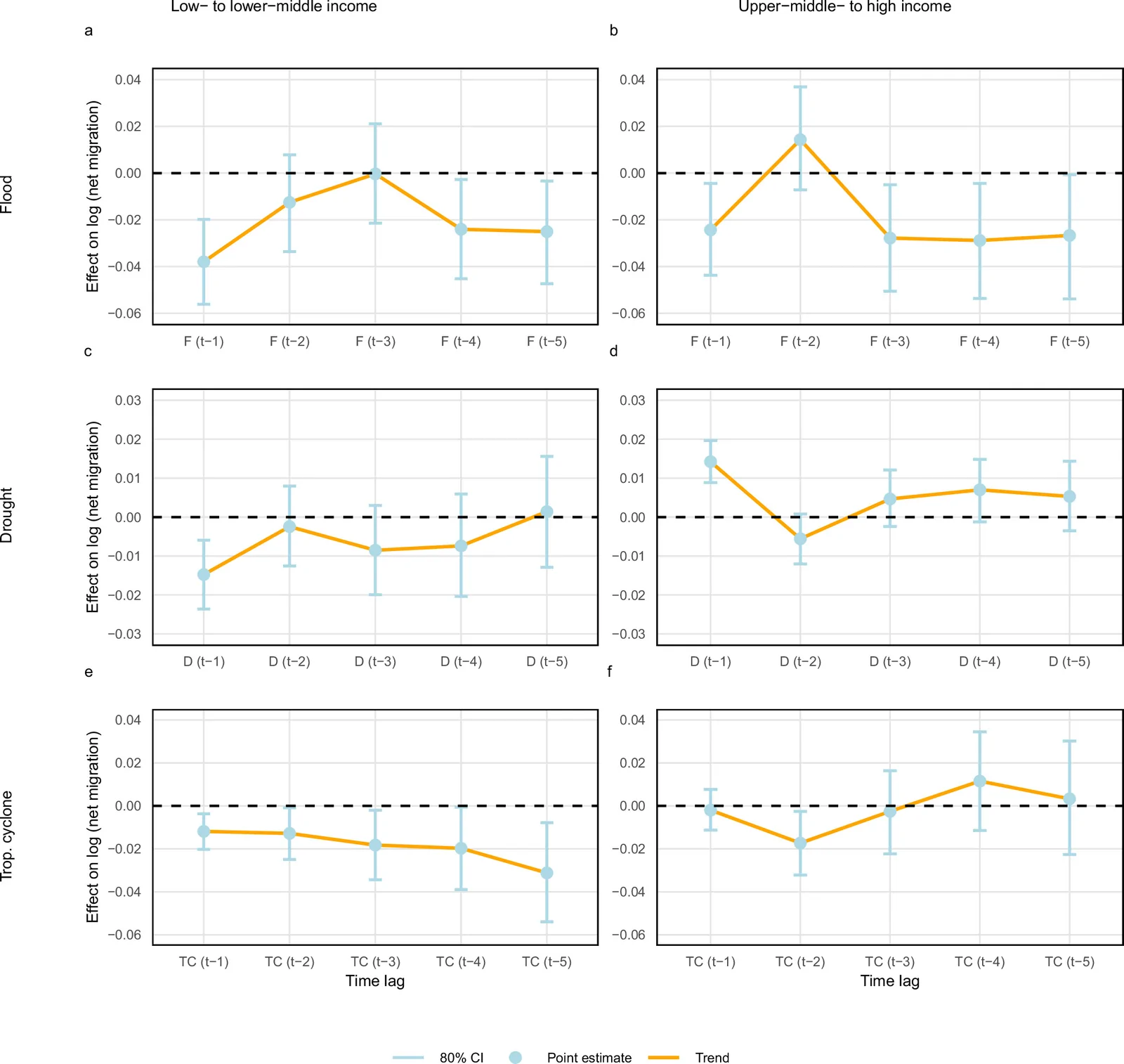
Associations between single climate hazards and net migration over a five-year lag in the positive net-migration sample, split by income group. Climate events (over a 5-year time lag) and net migration. Results show the associations between climate extremes and net migration in the positive net-migration sample, split by income groups: **a**, floods, low- to lower-middle-income countries (n = 34,660 district-year observations); **b**, floods, upper-middle- to high-income countries (n = 20,620 district-year observations); **c**, droughts, low- to lower-middle-income countries (n = 45,717 district-year observations); **d**, droughts, upper-middle- to high-income countries (n = 79,434 district-year observations); **e**, tropical cyclones, low- to lower-middle-income countries (n = 16,402 district-year observations); **f**, tropical cyclones, upper-middle- to high-income countries (n = 10,251 district-year observations). Coefficients are estimated using a hierarchical Bayesian panel vector autoregressive model (250,000 burn-in iterations, 250,000 post-burn-in iterations with thinning of 20), and error bars represent 80% credible intervals. Source data

We examine the associations between climate events and net migration with 1–5-year lags (Fig. 3). Full-sample results are provided in Supplementary Fig. 11, while the main text focuses on split samples for clearer interpretation. For flooding, in areas that usually lose population (negative net migration; Fig. 3a), net migration declines in the year immediately following flood exposure, with this negative effect persisting but gradually weakening over the next 4 years. In areas that typically attract people (positive net migration; Fig. 3b), flooding is linked to an immediate decline in net migration, which weakens temporarily before re-emerging as a significant negative effect 4–5 years later. The reason for this two-stage pattern is unclear. Although it could potentially reflect longer-term impacts on urban infrastructure or economic opportunities, we cannot rule out that this pattern may be influenced by data limitations or other factors not captured in our model. To examine heterogeneity, we split the sample by both income level (low-to-lower-middle versus upper-middle-to-high) and migration direction (Extended Data Figs. 2 and 3). In lower-income countries, flooding is associated with declines in net migration during the first 2 years in areas that typically lose population, and both immediately and 4–5 years later in areas that typically attract population. In higher-income countries, population-losing areas show declines 3 years after flooding, while population-attracting areas experience immediate and delayed negative associations across several years. The stronger immediate effects in lower-income settings possibly reflect greater vulnerability and fewer adaptation options, while higher-income settings may have more resources to delay or avoid migration following flood exposure.

When examining drought and net migration, we find that in districts with historically negative net migration (Fig. 3c), drought is associated with a decrease in net migration 2 years after exposure, while estimates at other time points remain uncertain. These areas, where people are more likely to move out, often face barriers such as weak infrastructure or limited economic opportunities that delay migration. Households may also need time to gather resources to move, especially under financial constraints^24^. By contrast, in areas with historically positive net migration (Fig. 3d), we observe a significant positive effect on net migration in the year right after drought, suggesting an initial inflow, although this pattern does not persist at later time lags. This pattern suggests that droughts may initially draw people to more economically dynamic areas where households are better able to adjust, although these effects are short-lived. For droughts, the timing and pattern of migration changes also differ by income level (Extended Data Figs. 2c,d and 3c,d). In areas that typically lose population, lower-income countries show no significant associations at any time point, while higher-income countries show a decline 2 years after drought. In areas that typically attract population, higher-income countries show a rise in net migration in the same period, while lower-income countries show an immediate decline. The delayed response in higher-income population-losing areas may reflect greater capacity for in-place adaptation, while this early increase in higher-income population-attracting areas may suggest that these locations attract population from surrounding regions. Lower-income population-attracting areas show immediate declines, possibly reflecting greater vulnerability and limited adaptive capacity.

For tropical cyclones, in areas with historically negative net migration (Fig. 3e), we find no clear associations at any time point. In areas with historically positive net migration (Fig. 3f), cyclones are associated with an immediate decline in net migration in the year following exposure, although this negative association weakens and becomes uncertain in subsequent years. This pattern is consistent with disruption to infrastructure, housing or economic activities that temporarily reduces the attractiveness of these areas, with conditions gradually stabilizing as recovery unfolds. The faster initial response may also reflect better connectivity to migration networks in these areas. For tropical cyclones, migration patterns differ by income level in both negative and positive net migration areas (Extended Data Figs. 2e,f and 3e,f). In areas that typically lose population, lower-income countries show a significant positive association 2 years after cyclone exposure, while higher-income countries show no significant associations at any time point. In areas that typically attract population, lower-income countries experience declines in net migration across all 5 years following cyclone exposure, while higher-income countries show a decline only 2 years after exposure. The persistent negative associations in lower-income population-attracting areas suggest that cyclones have sustained effects on the attractiveness or capacity of these areas to retain population. By contrast, the limited significant effects in higher-income settings may reflect greater capacity for recovery and reconstruction.

### Cumulative effects of repeated shocks

Finally, we expect that repeated exposure to the same hazard can shape migration patterns in ways that differ from those associated with single events. To explore such compounding relationships, we examine whether repeated exposure over time corresponds to different migration outcomes. Specifically, we assess whether migration patterns differ when climate extremes occur in consecutive years, reflecting the cumulative pressures of sustained climate stress. Details on the construction of the compound-event variables are provided in the Methods.

**Fig. 4:**
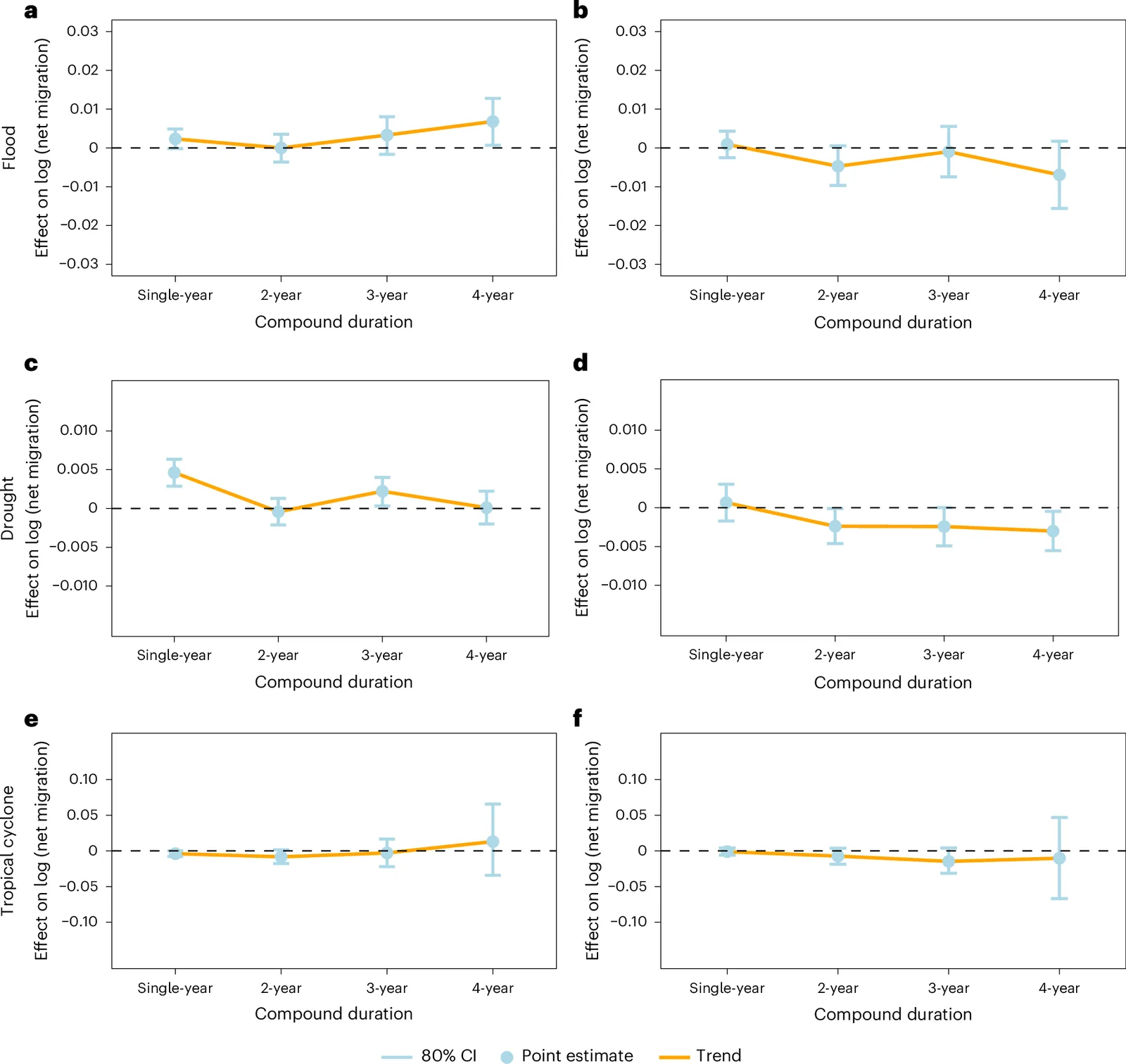
Compound climate events and net migration. Associations between repeated occurrences of the same type of climate extreme and net migration estimated using a hierarchical BPVAR model (12,500 burn-in iterations, 12,500 post-burn-in iterations with thinning of 20). The unit of study is the district–year observation. **a**–**f**, Sample sizes (*n*, district–year observations): floods, negative net migration: single-year (*n* = 165,860), 2-year (*n* = 80,140), 3-year (*n* = 43,240), 4-year (*n* = 29,500) (**a**); floods, positive net migration: single-year (*n* = 83,420), 2-year (*n* = 41,760), 3-year (*n* = 24,180), 4-year (*n* = 16,460) (**b**); droughts, negative net migration: single-year (*n* = 213,400), 2-year (*n* = 204,260), 3-year (*n* = 187,580), 4-year (*n* = 159,000) (**c**); droughts, positive net migration: single-year (*n* = 122,180), 2-year (*n* = 119,680), 3-year (*n* = 109,500), 4-year (*n* = 94,440) (**d**); tropical cyclones, negative net migration: single-year (*n* = 43,300), 2-year (*n* = 10,720), 3-year (*n* = 4,420), 4-year (*n* = 1,360) (**e**); tropical cyclones, positive net migration: single-year (*n* = 27,600), 2-year (*n* = 8,620), 3-year (*n* = 3,540), 4-year (*n* = 1,100) (**f**). Points show posterior mean coefficients and error bars represent 80% CIs. Supplementary Fig. 10 shows the prevalence of compound climate events over time. Source data

We first examine floods, where net migration responds differently to isolated versus consecutive events (Fig. 4a,b). In negative net migration areas (Fig. 4a), four consecutive years of flooding are associated with a significant positive effect on net migration, while shorter exposure durations show no significant associations. In positive net migration areas (Fig. 4b), no compound flood exposure pattern shows significant associations. These patterns differ from Fig. 3, where we found immediate negative effects of flooding at *t* − 1. The key difference is that Fig. 3 examines all floods regardless of whether they were preceded by earlier floods, whereas Fig. 4 separates isolated single-year floods from consecutive multi-year flooding sequences. These compound indicators are mutually exclusive where a district flooded in 2015, 2016, 2017 and 2018 is classified as experiencing a 4-year sequence in 2018. The positive association with four consecutive years therefore represents the total net migration response to this specific prolonged exposure pattern, not an additive effect of individual floods. This suggests that sustained flood exposure may reduce out-migration in population-losing areas relative to single flood, potentially reflecting adaptation in place or resource constraints under repeated stress. For drought (Fig. 4c,d), as with floods, we examine consecutive years of drought exposure rather than all droughts regardless of prior exposure. In negative net migration areas, single-year and 3-year exposures are associated with positive effects on net migration, whereas in positive net migration areas, sustained droughts of two or more consecutive years are associated with declines in net migration. The positive associations in population-losing areas may reflect constraints on mobility or adaptation in place under drought conditions relative to isolated droughts, whereas the negative associations in population-attracting areas indicate that prolonged drought erodes the capacity of these locations to attract or retain migrants relative to shorter drought sequences. This may reflect the cumulative effects of repeated drought stress, where initial coping strategies become depleted over time, leading to larger changes in migration patterns as drought persists^3^. For tropical cyclones (Fig. 4e,f), we find no significant associations for any compound exposure duration in either negative or positive net migration areas. Given the relatively few observations for extended cyclone sequences (Supplementary Fig. 10), we interpret these results with caution.

**Extended Data Fig. 4:**
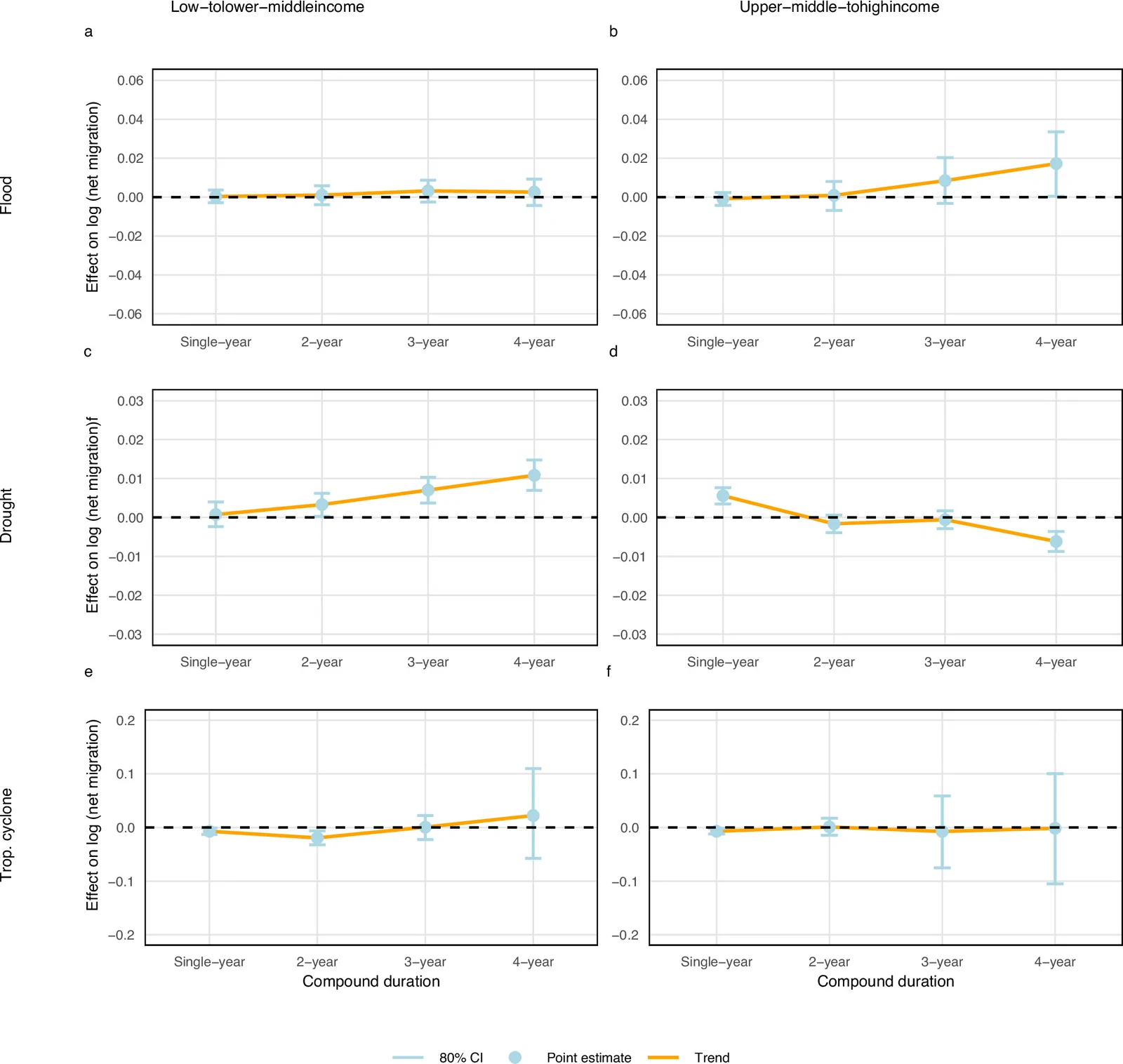
Associations between compound climate hazards and net migration in the negative net-migration sample, split by income group. Climate events (compound exposure) and net migration in the negative net-migration sample, split by income groups: low- to lower-middle-income (**a**, **c**, **e**) and upper-middle- to high-income countries (**b**, **d**, **f**), for floods (**a**-**b**), droughts (**c**-**d**) and tropical cyclones (**e**-**f**). Sample sizes (n, district-year observations): a, single-year (n=71,840), 2-year (n=44,779), 3-year (n=29,770), 4-year (n=22,040); **b**, single-year (n=76,521), 2-year (n=25,985), 3-year (n=9,925), 4-year (n=5,759); **c**, single-year (n=76,133), 2-year (n=70,305), 3-year (n=62,305), 4-year (n=54,639); **d**, single-year (n=124,048), 2-year (n=118,507), 3-year (n=106,598), 4-year (n=85,940); **e**, single-year (n=22,883), 2-year (n=7,238), 3-year (n=3,120), 4-year (n=600); **f**, single-year (n=17,957), 2-year (n=3,230), 3-year (n=1,290), 4-year (n=760). Coefficients are estimated using a hierarchical Bayesian panel vector autoregressive model (12,500 burn-in iterations, 12,500 post-burn-in iterations with thinning of 10), and error bars represent 80% credible intervals. Source data

**Extended Data Fig. 5:**
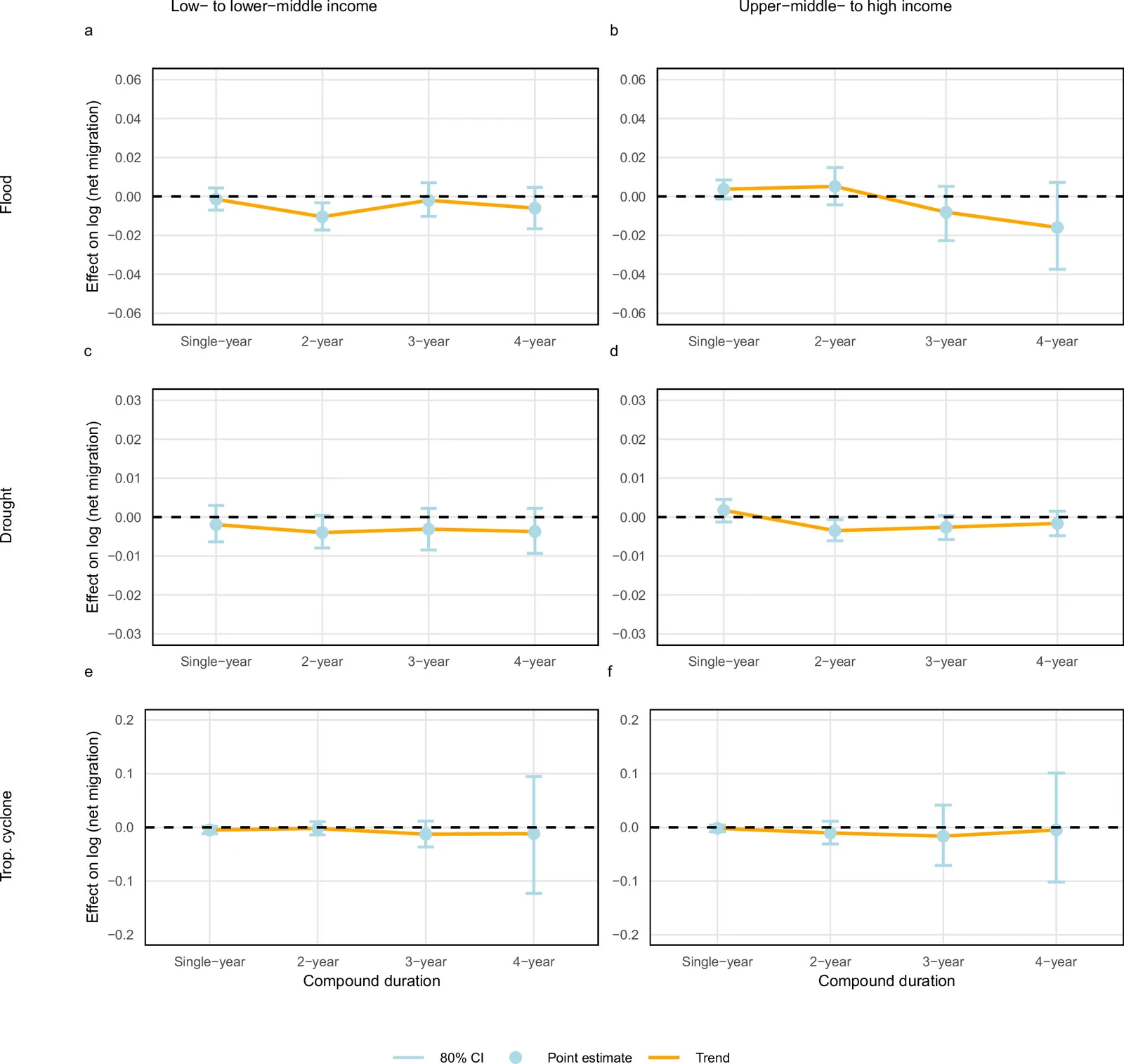
Associations between compound climate hazards and net migration in the positive net-migration sample, split by income group. Climate events (compound exposure) and net migration in the positive net-migration sample, split by income groups: low- to lower-middle-income (**a**, **c**, **e**) and upper-middle- to high-income countries (**b**, **d**, **f**), for floods (**a**-**b**), droughts (**c**-**d**) and tropical cyclones (**e**-**f**). Sample sizes (n, district-year observations): a, single-year (n=33,451), 2-year (n=23,727), 3-year (n=16,466), 4-year (n=11,940); **b**, single-year (n=42,400), 2-year (n=14,068), 3-year (n=5,836), 4-year (n=3,608); **c**, single-year (n=39,332), 2-year (n=37,489), 3-year (n=32,388), 4-year (n=28,012); **d**, single-year (n=77,018), 2-year (n=74,640), 3-year (n=68,643), 4-year (n=58,034); **e**, single-year (n=16,316), 2-year (n=6,217), 3-year (n=2,620), 4-year (n=440); **f**, single-year (n=10,527), 2-year (n=2,303), 3-year (n=870), 4-year (n=660). Coefficients are estimated using a hierarchical Bayesian panel vector autoregressive model (12,500 burn-in iterations, 12,500 post-burn-in iterations with thinning of 10), and error bars represent 80% credible intervals. Source data

When we split the analysis by income group (Extended Data Figs. 4 and 5), for floods, four consecutive years are associated with reduced out-migration in higher-income population-losing areas, while two consecutive years are associated with declines in lower-income population-attracting areas. For droughts, consecutive drought years are associated with reduced out-migration in lower-income population-losing areas across 2–4 years of exposure and mixed patterns in higher-income population-losing areas. In population-attracting areas, only higher-income settings show declines after two consecutive years. For tropical cyclones, population-losing areas show declines after single-year exposures in both income groups and after 2-year exposures in lower-income settings only, whereas population-attracting areas show no significant associations.

## Discussion

Our findings contribute to the literature on climate mobility by providing evidence on the associations between physical climate events and net migration, including both isolated and compound events. The patterns highlight how mobility responses vary by hazard type, migration context and income level. Migration responses unfold over different time horizons depending on the hazard and setting, with immediate effects for floods and cyclones alongside delayed drought responses. As such, non-governmental organizations and early warning systems can support informed mobility decisions by improving access to timely information on changing conditions.

For floods, exposure is associated with decreased net migration across contexts, with effects lasting several years and appearing stronger in lower-income areas. This pattern of reduced net migration in both population-losing and population-attracting areas suggests that floods both enhance out-migration and suppress in-migration, potentially owing to destruction of housing and infrastructure and heightened awareness of flood risk. These effects are consistent with constraints that limit sustained relocation in vulnerable contexts^37–39^. Four consecutive years of flooding are associated with reduced out-migration in population-losing areas relative to isolated floods, potentially reflecting adaptation in place or resource constraints under repeated stress, although short-term local benefits such as improved soil quality or reconstruction activity^40^ may also have a role.

For droughts, effects differ by income level and migration context: in lower-income areas, droughts are associated with decreased in-migration to population-attracting areas, potentially reflecting resource constraints^41^; in higher-income areas, droughts are associated with an amplification of existing migration patterns in both directions. Effects are generally short-term, consistent with financial constraints or limited mobility options that delay relocation in lower-income settings^24^. When droughts occur consecutively, sustained exposure of 2 years or more is associated with declines in population-attracting areas, which may indicate that prolonged drought erodes the capacity to attract migrants as coping strategies become depleted^3^.

For tropical cyclones, rapid-onset destructive events affect net migration primarily in lower-income settings, with effects lasting longer than might be expected for such acute disasters. In lower-income population-attracting areas, cyclones are associated with sustained declines in net migration across several years, potentially reflecting prolonged reconstruction efforts, whereas higher-income areas show minimal effects, possibly indicating faster recovery capacity. This is consistent with short-term disruption rather than persistent displacement^13,42–44^. For consecutive cyclone exposure, given the relatively few observations for extended cyclone sequences, we interpret these null results with caution.

Our study still faces several limitations. Annual net migration estimates combine two components: birth and death rates and the annual gridded population estimates of WorldPop. Birth and death rates change slowly and are relatively reliable at the district level. Estimates from WorldPop are the more critical input for capturing short-term migration, but they are interpolated between census counts rather than observed in most countries. Aggregating these gridded estimates to the district level improves their accuracy relative to the underlying grid cells. However, short-term associations remain sensitive to the quality of this downscaling. This limits our ability to confidently attribute same-year or single-year lag associations to specific climate events. Associations over longer horizons or compound exposures are less affected, as they average over periods less sensitive to short-term fluctuations in the underlying population estimates. This net migration measure, derived from the difference between total and natural population change, also prevents distinguishing among migration types or between internal and international flows. Net migration also conceals underlying volumes of in- and out-migration. Even when annual net migration is zero, areas may still experience substantial turnover, particularly during rapid-onset events. Because our measure captures only movements across administrative boundaries, it omits short-distance or intra-unit mobility, reflecting just a subset of overall movement. Detection of such mobility is also greater in countries with smaller administrative units. In that sense, both the ISIMIP hazard and downscaled migration data trade some spatial precision for global consistency. In addition, although our analysis captures average associations and income variation, it inevitably involves some overgeneralization. Unobserved heterogeneity such as political systems or rural development models may also shape climate–mobility relationships and should be explored further in future research. Finally, as with any observational design, our analysis identifies associations rather than causal effects. Although our models account for key socioeconomic and political factors, unobserved confounders such as adaptation measures or local migration systems may also influence the observed relationships. These findings should therefore be interpreted as indicative patterns that inform, rather than establish, causal relationships.

Research shows that climate events are associated with mobility changes unevenly between populations, with patterns differing by age, location, education, income and gender^45^. Addressing this diversity will be essential, particularly when analysing individual-level data or associations observed for compound events, to better understand who moves, who stays and why. Regions most exposed to climate extremes often also face armed conflict^46^, and the Intergovernmental Panel on Climate Change highlights that increasing extremes are linked with changes in mobility patterns^1^. Although single-country studies have shown that combining information on conflict and natural hazards can improve predictions of household mobility^47^, future research should examine how political instability and governance intersect with climate hazards at larger spatial scales and incorporate these dimensions into scenario modelling. Other hazards such as heatwaves, heavy rainfall and hailstorms may also influence mobility patterns and should be examined in future research.

## Methods

### Climate impact data

The climate data are made available by the ISIMIP^28^. ISIMIP data, standardized across several models and globally harmonized, allows for a comparable analysis of climate risks. However, this harmonization does not guarantee accuracy, as model outputs may still differ as a result of differences in structure, parameterization and simplified representations of local conditions such as crop systems. Despite these limitations, ISIMIP serves as a useful framework for examining the relationship between climate hazards and net migration across diverse contexts. We constructed four annual, population-weighted hazard indicators representing floods, droughts, tropical cyclones and crop failures, based on the ISIMIP 3b multi-model ensemble^28^. Each indicator is harmonized under a unified intensity–exposure framework and aggregated annually at the second administrative (Admin-2) level, corresponding to subnational districts (Admin-1 refers to provinces or states). Floods, droughts and tropical cyclones are expressed as population-weighted intensity–exposure measures, while crop failure is represented by a continuous negative deviation of annual yields from the decadal mean.

Flood data were obtained from the CaMa-Flood model^48^, which simulates daily river discharge and overbank flooding along a high-resolution global river network. The model outputs flood fraction data at 15 arcmin (≈25 × 25 km^2^) resolution, indicating the proportion of each grid cell inundated during events exceeding FLOPROS flood-protection thresholds^49^. To estimate population exposure, we combined CaMa-Flood outputs with gridded population data from the Global Human Settlement Layer (GHSL). GHSL data (30 arcsec, ≈1 km) for 1995, 2000, 2005, 2010, 2015 and 2020 were regridded to 15 arcmin, while preserving population sums and intermediate years were linearly interpolated. For each year, we multiplied flood fractions by the corresponding population counts in each grid cell, summed across the district and divided by total district population to obtain the population share exposed to flooding. This method accounts for the spatial concentration of flooding, typically in low-lying, river-adjacent areas, and aligns with approaches used in recent studies^50,51^. This procedure produces a standardized, population-weighted flood exposure indicator at Admin-2.

Droughts, available at 30 × 30 arcmin^2^ resolution (approximately 0.5^∘^; ≈50 × 50 km^2^), are measured as the number of months in a year in which the soil moisture in the top soil layer (0–100 cm) is equal to or lower than the 10th percentile of the historical monthly distribution from 1901 to 2019. The ensemble includes the models CLASSIC, CWatM, H08, JULES-W2, MIROC-INTEG-LAND, ORCHIDEE-MICT, WaterGAP2-2e and WEB-DHM-SG. To ensure comparability with other hazard indicators, we converted this variable into a population-weighted intensity–exposure measure. Drought intensity, defined as the number of drought months per year divided by 12 (ranging from 0 to 1), was combined with gridded population data from the GHSL to account for exposure. The resulting indicator captures both the duration and population exposure to drought conditions.

Tropical cyclones, available at a 5 arcmin (≈0.083^∘^; ~10 × 10 km^2^) resolution, are defined as grid cells experiencing maximum wind speeds exceeding 34 knots. The data are derived from the International Best Track Archive for Climate Stewardship and include all recorded tropical cyclones from 1950 to 2021^28,52^. We identified all grid cells with wind speeds ≥34 knots and computed the population share exposed within each district.

Finally, crop failure, available at a 30 arcmin (0.5^∘^; ≈50 × 50 km^2^) resolution, is derived from combined annual yields of maize, rice, soy and wheat, based on an ensemble of global crop models (ACEA, CROVER, CyGMA1p74, ISAM, LDNDC, LPJmL, pDSSAT and PEPIC)^53^. We calculate a continuous yield anomaly as the negative deviation of annual yields from the 10-year moving average after detrending long-term growth. This continuous metric captures the magnitude of yield shortfalls, rather than treating crop failure as a binary occurrence, improving cross-hazard comparability under the unified intensity–exposure framework. All climate events are aggregated to Admin-2 district and year as our unit of analysis. Supplementary Fig. 1 visualizes cumulative hazard values by district over the study period. Boundaries of Admin-2 areas are made available through the Database of Global Administrative Areas version 4.1^54^.

### Net migration data

The net migration dataset provides annual estimates for 2000–2019 at approximately 10-km resolution^29^. It was developed by combining gridded population data from WorldPop with downscaled estimates of natural population change, derived from harmonized subnational birth and death records covering over 2,500 administrative units globally. Missing data were addressed through administrative boundary harmonization, interpolation using regional or neighbouring trends and alignment with national totals. The cleaned data were downscaled using multiple linear regression and area-to-point kriging, incorporating covariates such as population density, human development, the share of women of reproductive age and the ratio of average age to life expectancy. Net migration was then calculated as the difference between observed and natural population change per grid cell. The estimates were validated against subnational data from Europe, the United States and South Korea and national data from the United Nations. Although this approach captures broad spatial patterns, it cannot distinguish between migration types (for example, internal versus international) or very short-term displacements. Nonetheless, it enables globally consistent, subnational analyses of migration trends over time.

#### Covariates

Conflict intensity was measured as number of battle-related deaths for three types of conflict—intrastate armed conflict, non-state conflict and one-sided violence at Admin-2 level as per the definition of the Uppsala Conflict Data Program^30,31^. Intrastate armed conflicts concern an incompatibility regarding either territory (including secession or demands for autonomy) or government (including change of the incumbent government or the current political regime)^55^. Intrastate conflicts are often referred to as civil conflicts or civil wars, fought between a state and its adversaries—a formally organized non-state opposition group. Formal groups are understood here as “any non-governmental group of people having announced a name for their group and using armed force”^55^. According to the definition provided by the Uppsala Conflict Data Program, this form of state-based conflict is an armed clash that reaches at least 25 battle-related deaths annually^30,55^. Armed non-state conflict, defined as fighting between non-state groups, without the direct involvement of the state government is also a common phenomenon. Finally, one-sided violence refers to the the use of armed force by the government of a state or by a formally organized group against civilians which results in at least 25 deaths.

Ethnic discrimination was measured as the size of an ethnic group, residing in an Admin-2 unit, which is categorized as discriminated against or powerless by the Ethnic Power Relations (EPR) core dataset^32^. Subnational Human Development Index is an average of the subnational values of three dimensions: education, health and standard of living, available at the Admin-1 unit^35^. Economic activity levels was measured as log gross domestic product per capita per Admin-2 level^34^. Population size is the log-transformed absolute number of population per Admin-2 level^36^. Liberal democracy was measured as the extent to which a country is considered a liberal democracy, provided by the Variety of Democracy Project^33^. Finally, low-income subsample countries are defined as those classified under the World Bank income groups, low income and lower-middle income. High-income subsample countries include those classified as upper-middle income and high income, on the basis of the income groupings of the World Bank.

### Mixed-effects regression model

We estimated mixed-effects linear regressions with random intercepts for country and year to account for unobserved spatial and temporal heterogeneity. To capture structural differences in migration dynamics, we classified districts on the basis of the direction of their net migration rate over time. A district was categorized as having negative net migration if its annual rate was negative in at least 15 of the 20 years (2000–2019) and as positive net migration if the rate was positive in at least 15 of the 20 years. This rule identifies areas with consistent in- or out-migration patterns regardless of magnitude. Supplementary Fig. 2 shows the underlying distribution used to define these groups. Descriptive statistics for the right-hand side variables are presented in Supplementary Table 1. Additionally, Supplementary Fig. 2 provides density plots for these variables, while Supplementary Figs. 3.1 and 3.2 illustrates their trends over time. The correlation matrix in Supplementary Fig. 6 shows the absence of multicollinearity, as none of the variables are highly correlated with each other.

### Model fit evaluation

In addition to our regression analysis, we conducted a model fit evaluation using the AIC to compare six different model specifications and explore which factors and combinations most effectively predict patterns of net migration. Supplementary Fig. 9a shows the performance of each model on the *y* axis from top to bottom. Our full climate model, which includes all climate extremes and sociopolitical variables (climate variables + socioeconomic and political variables (SEP)), outperforms the others, highlighting its superior predictive capability in explaining variation in net migration. Examining Supplementary Fig. 9a from left to right, the smallest differences in AIC values occur between the full climate model and those that include individual climate and sociopolitical variables (tropical cyclone + SEP, flood + SEP, drought + SEP). Since smaller AIC differences indicate closer model performance, this suggests that the full climate model is the best-performing specification, followed by the tropical cyclone, flood and drought models. Lastly, the base model, which includes only SEP, shows the weakest fit, as indicated by the darker blue colours on the right side of the matrix, which represent larger differences in AIC values.

Additionally, we performed an out-of-sample 100-fold cross-validation, calculating the root mean square error (RMSE.) to further validate the robustness and generalizability of the models across different data splits, ensuring that the results are not dependent on a specific partitioning of the dataset^56^. To evaluate the out-of-sample predictive performance of our models, we partition our main dataset into two sets: a training set, used to fit the models, and a test set, used to evaluate the ability of the model to predict the dependent variable on unseen data. Specifically, we assess the predictive performance by iteratively defining 70% of the data as the training set, fitting the model and then evaluating its performance on the remaining 30% left-out subsample. This process is repeated 100 times and the overall predictive performance is assessed by aggregating the prediction errors across all iterations^57^. We use the RMSE as the measure of error, calculated as the square root of the average squared differences between observed and predicted values. This cross-validation approach allows for an assessment of the predictive accuracy of the model and provides a robust test of the contribution of each climate extreme event in predicting net migration for new, unseen observations, while safeguarding against overfitting concerns^58^. It ensures that the model generalizes well to unseen data and that the evaluation is robust and not biased by any single data split. Consistent with the AIC criterion, the out-of-sample validation in Supplementary Fig. 9b shows that the flood model and the fully specified model including all climate variables exhibit the highest performance, indicated by the smallest RMSE.

Mixed-effect linear regression model analyses panel data with a random intercept for year and country. The random year and country effects account for cluster-confounding issue by modelling the two levels in the data:countries and years as the higher grouping structure and districts, nested in the level 2 variables as the lower units^59^. The random year and country intercepts account for unobserved historical, political or socioeconomic characteristics that districts within the same country share, which could make them more likely to be similar to each other than to districts in other countries^60^. Overall, this multilevel approach allows for modelling the variation of district-level regression coefficients across groups and therefore a range of unmeasured social processes, which might include the omitted variables themselves^61^. We estimate the following equation.

1 $$\begin{array}{ll}{Y}_{ijt}\;=\, {\beta }_{0}+{\beta }_{1}{Y}_{ijt-1}+{\beta }_{2}{\mathrm{Flood}}_{ijt}+{\beta }_{3}{\mathrm{Drought}}_{ijt}+{\beta }_{4}{\mathrm{TC}}_{ijt}\\\qquad \,{\beta }_{6}\log ({\mathrm{AC}}_{ijt-1})+{\beta }_{7}\log (\mathrm{NC}_{ijt-1}) \,+{\beta }_{8}\log (\mathrm{OSV}_{ijt-1})\\\qquad+{\beta }_{9}{\mathrm{LD}}_{ijt-1}+{\beta }_{10}\log ({\mathrm{Pop}}_{ijt-1}) \,+{\beta }_{11}{\mathrm{ED}}_{ijt-1}\\\qquad+{\beta }_{12}\log (\mathrm{GDPpc}_{ijt-1})+{\beta }_{13}{\mathrm{SHDI}}_{ijt-1} \,+{u}_{i}+{v}_{t}+{\upepsilon }_{ijt}\end{array}$$

In this equation, *Y*_*ijt*_ represents log-transformed net migration at the Admin-2 and year level. The intercept is denoted by *β*_0_, while *u*_*i*_ and *v*_*t*_ represent the random effects for country *i* and year *t*, respectively. The error term is indicated by *ϵ*_*ijt*_. The remaining terms are the predictors used in the model: tropical cyclone (TC), armed conflicts (AC), ethnic discrimination (ED), liberal democracy (LD), non-state conflicts (NC), population size (Pop).

#### Hierarchical Bayesian panel vector autoregressive model

To examine short- and longer-term effects of climate extremes on net migration, we apply a hierarchical BPVAR model, a framework commonly used for multivariate time series analysis. Unlike standard regression models, which assume independent observations and unidirectional relationships, the panel BVAR model accounts for dynamic interdependencies by allowing all variables to influence each other over time within each district, while estimating average effects across districts through hierarchical modelling that accounts for both shared patterns and local variation. Each variable is modelled as a function of its own lagged values and those of other variables, capturing feedback mechanisms and delayed responses. The Bayesian hierarchical approach incorporates prior information to stabilize estimates and borrows strength across districts, particularly useful when working with thousands of subnational units with varying data quality, and regularizes coefficients to prevent overfitting.

We estimate panel BVARs separately for each climate hazard (floods, droughts and tropical cyclones). Before estimation, both net migration and the climate variable are globally standardized using means and standard deviations from the full sample. Time-varying socioeconomic and political controls are incorporated as exogenous variables and similarly standardized, ensuring that the BPVAR captures dynamic responses to climate shocks net of confounding trends. The model is estimated using the bpvars package in R, which implements hierarchical priors that allow district-specific coefficients to vary around global means^62^. For the hazard models, we draw posterior samples after a burn-in period, with thinning applied to reduce autocorrelation between draws, to approximate the posterior distribution of parameters. Exact iteration counts for each model are reported in the corresponding figure captions. This approach yields posterior means and CIs that reflect parameter uncertainty. We report global coefficients that represent average migration–climate effects across all districts.

We estimate a BVAR model of the form:

2 $${Y}_{ijt}={\bf{A}}_{0}+\mathop{\sum }\limits_{\ell =1}^{5}{A}_{\ell }{{\bf{y}}}_{t-\ell }+C{{\bf{x}}}_{t}+{{\bf{u}}}_{t},$$

where:

- *Y*_*ijt*_ is the log-transformed net migration at time *t* for district *j* in country *i*.
- **y**_*t*−*ℓ*_ denotes the vector of endogenous variables at lag *ℓ*. In practice, the model is estimated separately for each climate hazard, so that **y**_*t*−*ℓ*_ consists of log net migration and a single climate variable (flood, drought or tropical cyclone) measured at time *t* − *ℓ*.
- **x**_*t*_ is a vector of time-varying socioeconomic and political variables, including armed conflict, non-state conflict, one-sided violence, democracy, population size, ethnic group size, GDPpc and the subnational human development index.
- **A**_0_ is the intercept term.
- *A*_*ℓ*_ are coefficient matrices associated with the *ℓ*th lag of the endogenous variables, for *ℓ* = 1, …, 5.
- *C* is the coefficient matrix associated with the exogenous variables.
- **u**_*t*_ is the vector of error terms, assumed to follow a multivariate normal distribution, ${{\bf{u}}}_{t} \sim {\mathcal{N}}(0,\Sigma )$.

The hierarchical structure of the panel BVAR estimates district-specific coefficients for each parameter (**A**_0_, *A*_*ℓ*_, *C*) while imposing a common prior that allows these coefficients to vary around global means. This partial pooling addresses heterogeneity in data quality across districts: estimates for districts with limited observations are regularized towards the global average, preventing overfitting, whereas districts with extensive data are permitted to deviate from the global pattern when the data strongly support such differences.

#### Compound climate-event variables

To operationalize compounding exposure, we construct binary indicators capturing sequences of repeated events of the same hazard type across consecutive years. To construct these indicators, the hazard intensity measures are first converted into binary annual exposure variables. A district is coded as exposed in a given year if the population-weighted hazard measure is strictly greater than zero and as unexposed otherwise. Compound indicators then track uninterrupted sequences of these annual binary exposures. Specifically, compound events are defined as mutually exclusive categories on the basis of the length of uninterrupted exposure. A single-year event is coded as 1 if a hazard occurs in year *t* but not in *t* − 1. A 2-year compound event is coded as 1 if the same hazard occurs in both *t* and *t* − 1, but not in *t* − 2. This logic extends to longer sequences: 3-year compound events require uninterrupted exposure over *t* to *t* − 2 with no event in *t* − 3 and 4-year compound events require exposure over *t* to *t* − 3 with no event in *t* − 4. All compound variables were generated separately for floods, droughts and tropical cyclones, following the same population-weighted constructs described earlier.

### Reporting summary

Further information on research design is available in the Nature Portfolio Reporting Summary linked to this article.

## Online content

Any methods, additional references, Nature Portfolio reporting summaries, source data, extended data, supplementary information, acknowledgements, peer review information; details of author contributions and competing interests; and statements of data and code availability are available at https://doi.org/10.1038/s41558-026-02752-4.

## Supplementary information

Supplementary Information Supplementary Figs. 1–12 and Tables 1–4.

Reporting Summary

Supplementary Data Source data for Supplementary Figs. 7–12.

## Source data

Source Data Fig. 2 Statistical source data.

Source Data Fig. 3 Statistical source data.

Source Data Fig. 4 Statistical source data.

Source Data Extended Data Fig. 1 Statistical source data.

Source Data Extended Data Fig. 2 Statistical source data.

Source Data Extended Data Fig. 3 Statistical source data.

Source Data Extended Data Fig. 4 Statistical source data.

Source Data Extended Data Fig. 5 Statistical source data.

## Data Availability

All processed datasets necessary to reproduce the findings of this study are available via Zenodo at https://doi.org/10.5281/zenodo.21843679 (ref. ^63^) and are also available via GitHub at https://github.com/kpppetr/district-migration-climate-impacts. The repository contains the harmonized district-level migration and climate exposure data used in the analyses, along with documentation of data sources and processing steps. All third-party input datasets are publicly accessible through their respective providers, as cited in the references. No restrictions apply to the availability of the processed replication datasets. Source data are provided with this paper.

## Extended data

**Extended Data Table 1:** District (Admin-2) fixed-effects regression estimates of the association between climate hazards and net migration

Variable	Net Migration	Negative Net Migration	Positive Net Migration
log(net migration) (t-1)	−0.212***	−0.145***	−0.069***
(SE)	0.002	0.004	0.007
Flood	0.022	−0.092	0.268*
(SE)	0.100	0.123	0.145
Drought	0.004	0.037	−0.041
(SE)	0.062	0.074	0.089
Trop cyclone	−0.030	−0.027	−0.038**
(SE)	0.019	0.021	0.018
log(armed conflict brd) (t-1)	0.013	0.001	−0.011
(SE)	0.008	0.012	0.012
log(non-state conflict brd) (t-1)	0.016	0.020	0.001
(SE)	0.014	0.021	0.022
log(OSV conflict brd) (t-1)	0.004	0.017	−0.042.
(SE)	0.014	0.021	0.023
Liberal democracy (t-1)	0.198***	0.285***	−0.352***
(SE)	0.046	0.057	0.107
log(population) (t-1)	−0.470***	−0.205***	−0.763***
(SE)	0.018	0.037	0.046
Ethnic discrimination (t-1)	0.345***	0.158.	0.236**
(SE)	0.064	0.094	0.113
GDP pc (t-1)	0.108***	0.100***	0.045
(SE)	0.015	0.021	0.030
SHDI index (t-1)	−2.150***	−0.830***	−0.227
(SE)	0.164	0.260	0.404
Observations	536.795	191.236	115.951
RMSE	1.802	1.255	1.074
Adj. R2	0.442	0.280	0.365
Within R2	0.048	0.023	0.025

Regression coefficients, standard errors and significance levels from district (Admin-2) fixed-effects regressions of log net migration on floods, droughts and tropical cyclones, together with conflict, governance and socioeconomic controls, for the full sample and split by negative and positive net-migration areas. Corresponding coefficient plots are shown in Extended Data Fig. 1.

## Code availability

All code used for data processing, statistical analysis and visualization is available via GitHub at https://github.com/kpppetr/district-migration-climate-impacts. The analysis was conducted in R (version 4.4.3) and the repository includes all scripts and package specifications required to reproduce the results.
