# Supplementary material for: Compound and heterogeneous relationships between climate extremes and global net migration
Source: Nat Clim Chang. 2026 Sep 11;16(10):1162–9. doi: 10.1038/s41558-026-02752-4 (PMC13638451; doi:10.1038/s41558-026-02752-4)
Supplement: Supplementary file 1 [file 41558_2026_2752_MOESM1_ESM.pdf]

nature climate change

Supplementary information

https://doi.org/10.1038/s41558-026-02752-4

Compound and heterogeneous relationships between climate extremes and global net migration

In the format provided by the authors and unedited

Supplementary Information: Compound and heterogeneous relationships between climate extremes and global net migration

1 Descriptive statistics

Table S.1: Descriptive statistics for all variables included in the model specification. Summary includes mean, standard deviation, minimum, and maximum values.

Statistic	Min	Pctl(25)	Median	Mean	Pctl(75)	Max	St. Dev.
Log (net migration)	−12.177	−2.466	−0.945	−0.347	1.960	13.059	2.469
Floods	0.000	0.000	0.000	0.005	0.000	0.920	0.037
Drought	0.000	0.000	0.001	0.027	0.028	1.000	0.058
Tropical Cyclones	0.000	0.000	0.000	0.020	0.000	1.000	0.133
Crop failure	0.000	0.000	0.000	0.033	0.000	1.000	0.159
Log Armed Conflict Brd	0.000	0.000	0.000	0.041	0.000	8.799	0.368
Log Communal Conflict Brd	0.000	0.000	0.000	0.008	0.000	8.067	0.158
Log One-sided Violence Brd	0.000	0.000	0.000	0.014	0.000	7.962	0.196
Human Development Index	0.183	0.657	0.734	0.725	0.818	0.987	0.138
Ethnic Group Size	0.000	0.000	0.000	0.074	0.044	1	0.158
Log Population	0.000	9.278	10.430	10.436	11.574	17.255	1.784
Log GDP pc	5.037	8.381	9.143	9.161	10.125	13.531	1.113
Log Area	0.778	5.072	6.430	6.438	7.567	13.834	1.703
Liberal Democracy Index	0.005	0.251	0.494	0.497	0.768	0.898	0.264

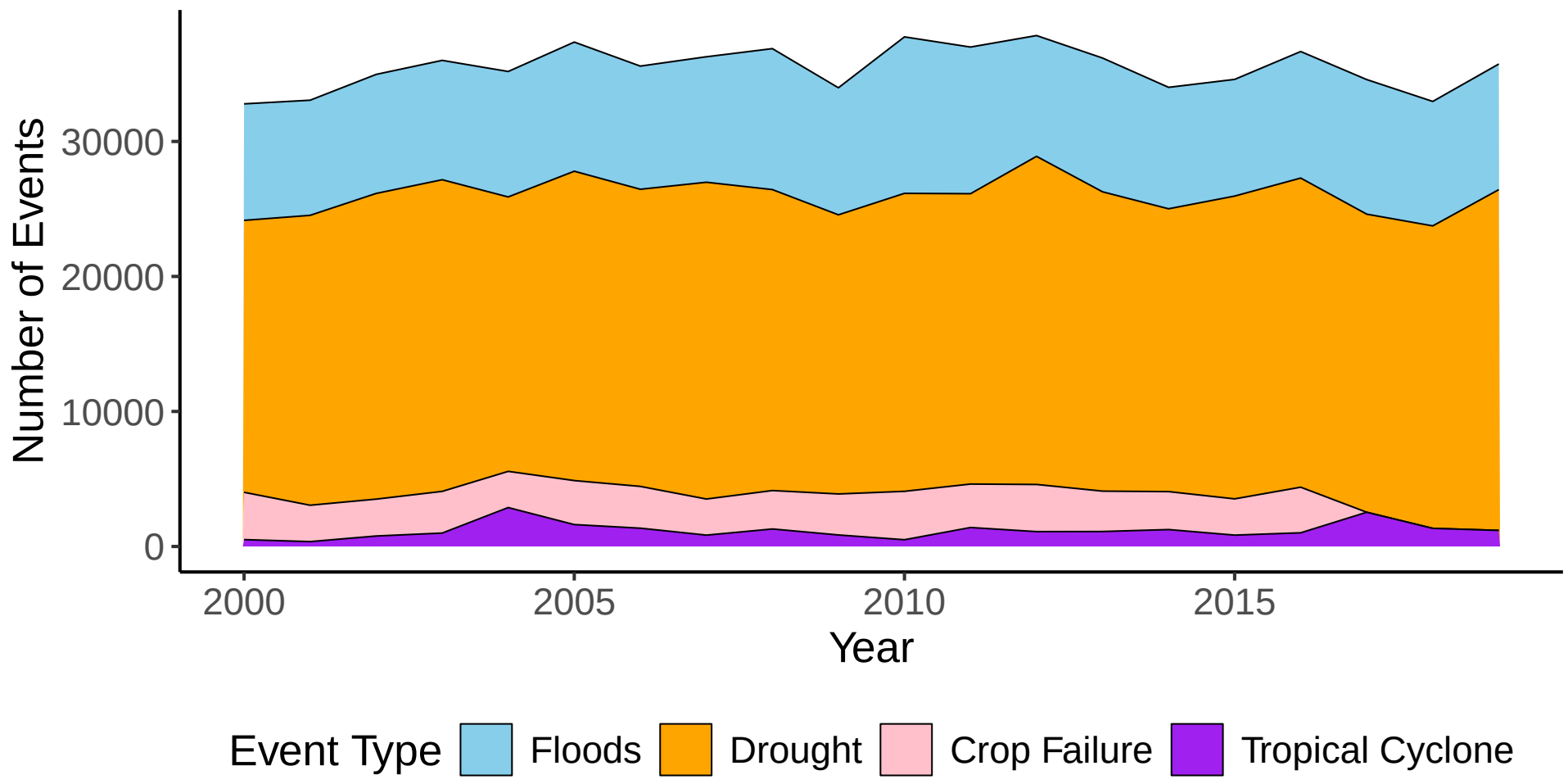

Figure S.1: Total number of extreme climate events during the studied period per district and year
.

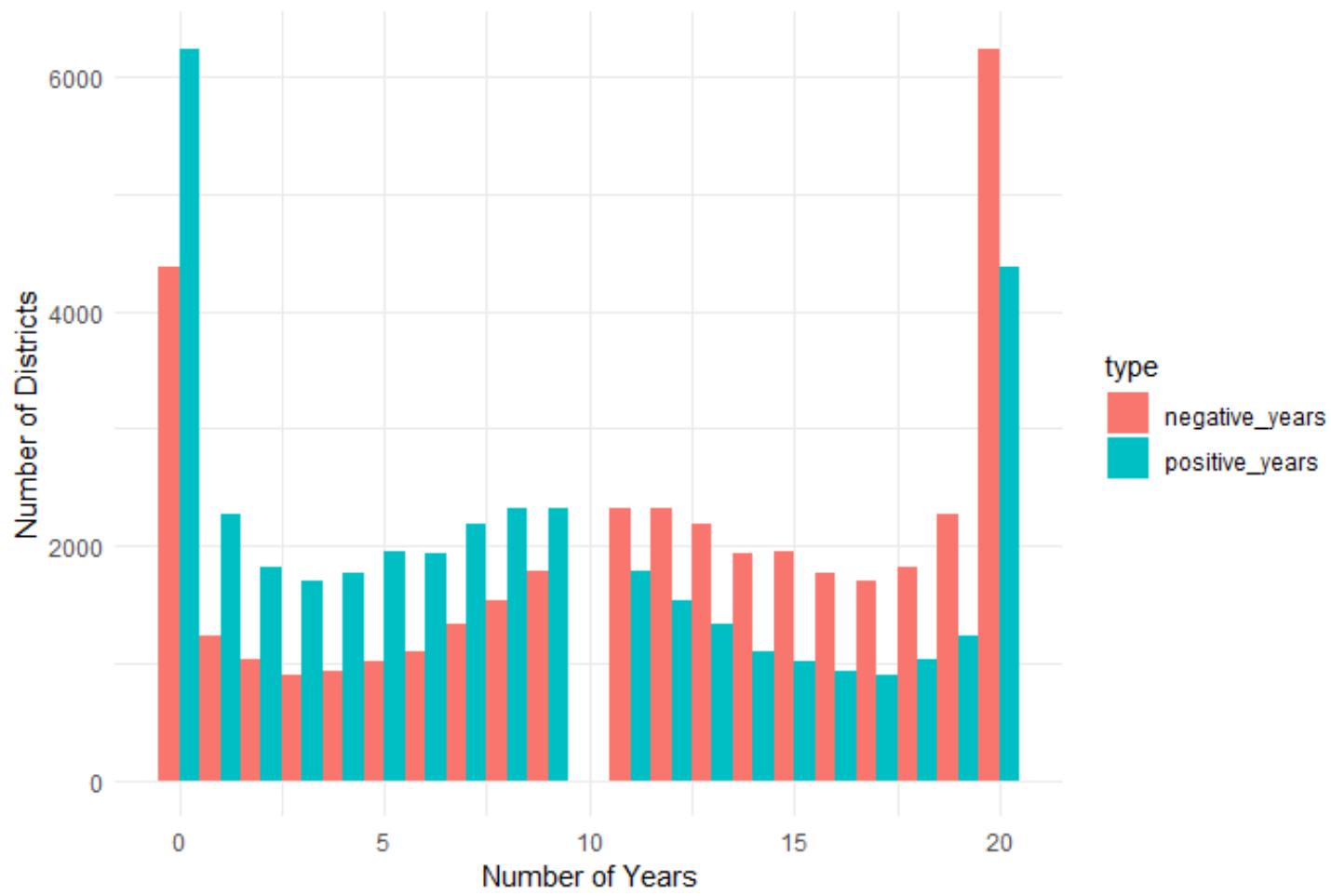

Figure S.2: Histogram, illustrating the number of districts categorized by the number of years they experienced positive or negative net migration. Districts with more than 10 years of positive or negative net migration are highlighted, showing the frequency of sustained migration trends across different regions.
.

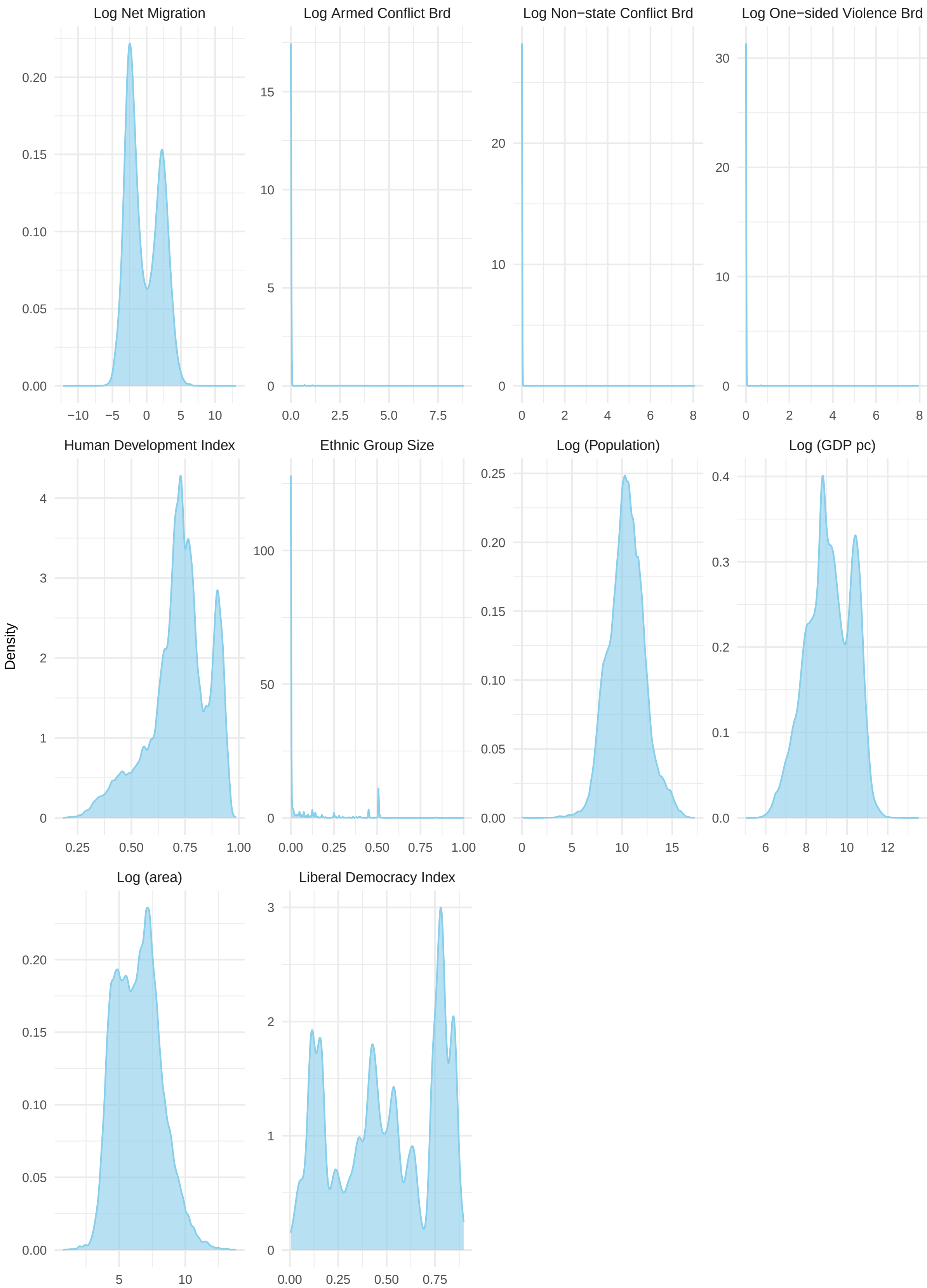

Figure S.3.1: Density plots for key control variables

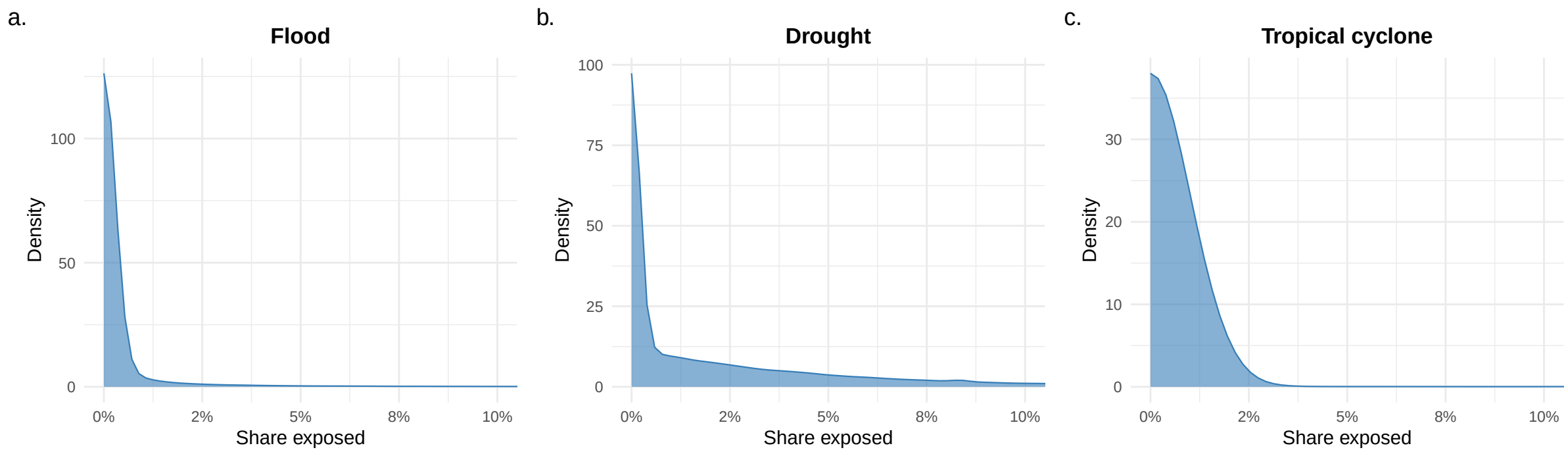

Figure S.3.2: Density plots for key climate variables

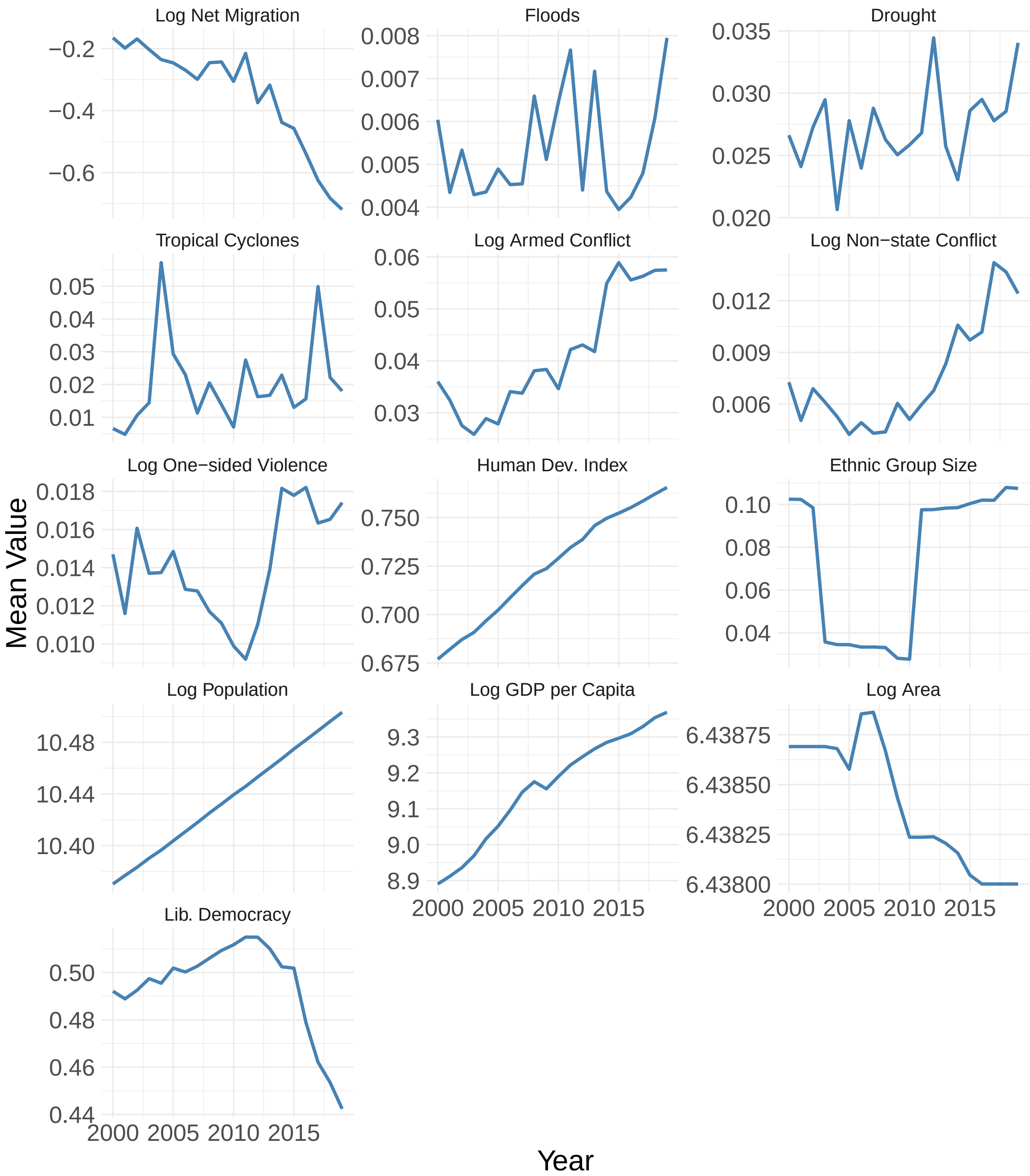

Figure S.4: Annual trends averaged across all districts for key variables over the study period

2 Robustness

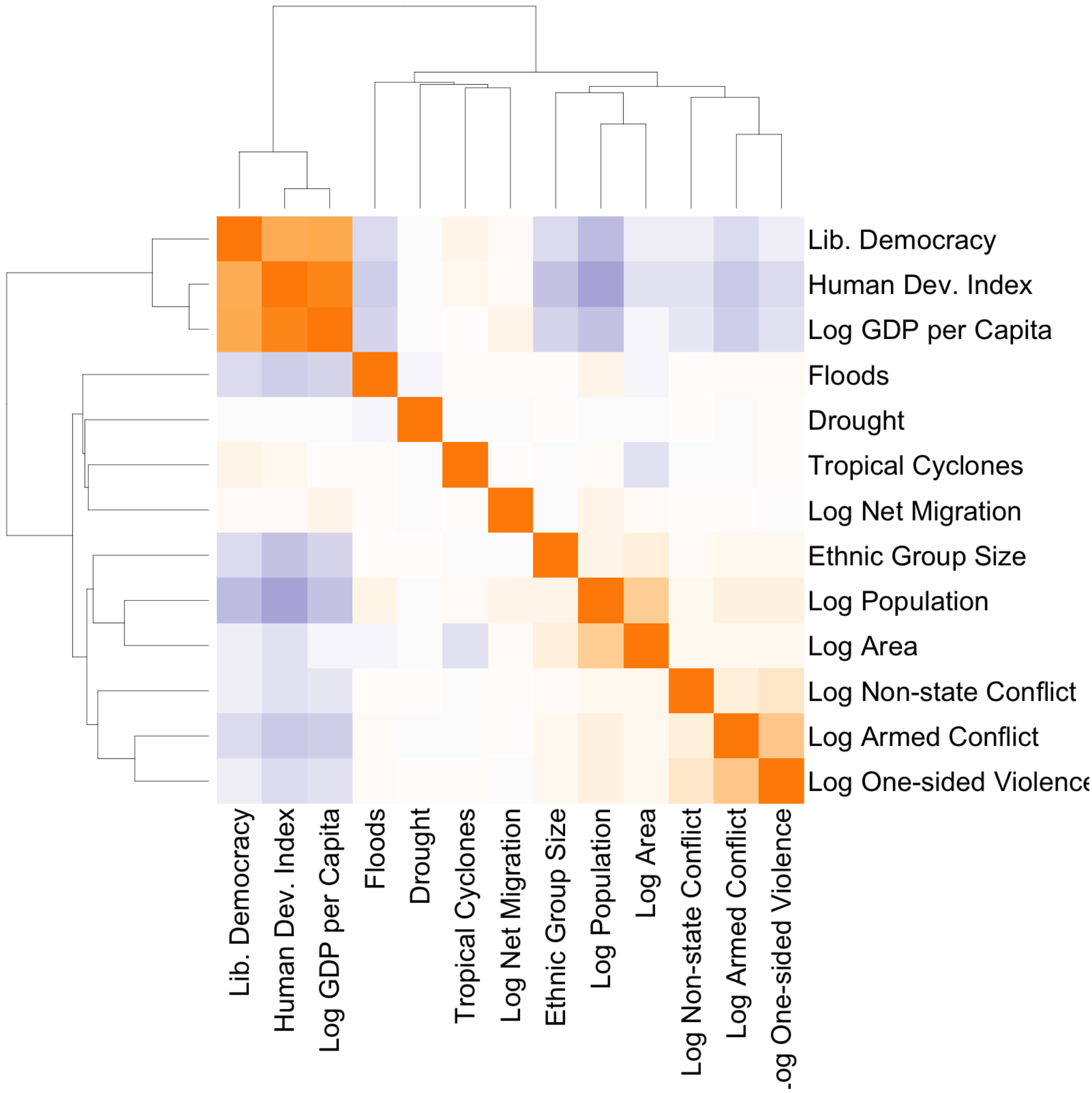

Figure S.6: Correlation matrix for right-hand side variables, significance level at 1%;

.

2.1 Random effect regression tables

Table S.2: Global Analysis of Single Climate Events and Net Migration. Results show the effect of climate extremes on net migration, for the full sample, negative net migration, and positive net migration, displaying coefficients for mixed effects linear regression with random intercepts for year and country.

	Dependent variable:		
	Net Migration	Negative Net Migration	Positive Net Migration
	(1)	(2)	(3)
log(net migration) ($t-1$)	0.251***	0.085***	0.273***
	(0.001)	(0.002)	(0.003)
Flood	0.288***	−0.012	0.426***
	(0.093)	(0.100)	(0.119)
Drought	−0.077	0.059	−0.066
	(0.053)	(0.057)	(0.076)
Trop cyclone	0.084***	0.128***	−0.080***
	(0.023)	(0.027)	(0.026)
log(armed conflict brd) ($t-1$)	−0.021**	−0.089***	−0.002
	(0.009)	(0.011)	(0.011)
log(non−state conflict brd) ($t-1$)	0.018	−0.012	−0.006
	(0.020)	(0.021)	(0.022)
log(OSV conflict brd) ($t-1$)	0.012	0.029	−0.028
	(0.017)	(0.018)	(0.019)
Liberal democracy ($t-1$)	0.155***	0.254***	−0.511***
	(0.056)	(0.052)	(0.072)
log(population) ($t-1$)	0.120***	0.108***	0.006*
	(0.003)	(0.003)	(0.003)
Ethnic discrimination ($t-1$)	0.164***	−0.106**	0.114**
	(0.043)	(0.045)	(0.057)
GDP pc ($t-1$)	0.262***	−0.024***	0.130***
	(0.008)	(0.009)	(0.009)
log(area)	0.050***	0.010***	0.004
	(0.003)	(0.004)	(0.003)
SHDI index ($t-1$)	2.352***	1.231***	−0.750***
	(0.090)	(0.099)	(0.105)
Observations	528,250	189,887	111,467
Akaike Inf. Crit.	2,380,895.000	674,204.900	372,018.700
Bayesian Inf. Crit.	2,381,085.000	674,377.600	372,182.200
Note:			*p<0.1; **p<0.05; ***p<0.01

2.2 Fixed effect model

Table S.3: Fixed Effects OLS Models. Results show the effect of climate extremes on net migration for the full sample, negative net migration, and positive net migration, using OLS with country and year fixed effects.

	Dependent variable:		
	Net Migration	Negative	Positive
	(1)	(2)	(3)
log(net migration) ($t-1$)	0.251***	0.084***	0.272***
	(0.001)	(0.002)	(0.003)
Flood	0.280***	−0.014	0.428***
	(0.093)	(0.100)	(0.120)
Drought	−0.078	0.059	−0.061
	(0.053)	(0.057)	(0.076)
Trop cyclone	0.083***	0.127***	−0.079***
	(0.023)	(0.027)	(0.026)
log(armed conflict brd) ($t-1$)	−0.021**	−0.089***	−0.002
	(0.009)	(0.011)	(0.011)
log(non−state conflict brd) ($t-1$)	0.018	−0.012	−0.005
	(0.020)	(0.021)	(0.022)
log(OSV conflict brd) ($t-1$)	0.012	0.030*	−0.030
	(0.017)	(0.018)	(0.019)
Liberal democracy ($t-1$)	0.215***	0.262***	−0.553***
	(0.057)	(0.055)	(0.082)
log(population) ($t-1$)	0.120***	0.108***	0.007**
	(0.003)	(0.003)	(0.003)
Ethnic discrimination ($t-1$)	0.167***	−0.098**	0.117**
	(0.044)	(0.045)	(0.058)
GDP pc ($t-1$)	0.265***	−0.023***	0.131***
	(0.008)	(0.009)	(0.009)
log(area)	0.051***	0.011***	0.004
	(0.003)	(0.004)	(0.003)
SHDI index ($t-1$)	2.486***	1.300***	−0.716***
	(0.092)	(0.105)	(0.115)
Country FE	Yes	Yes	Yes
Year FE	Yes	Yes	Yes
Observations	528,250	189,887	111,467
R^2	0.143	0.123	0.155
Note:			*p<0.1; **p<0.05; ***p<0.01

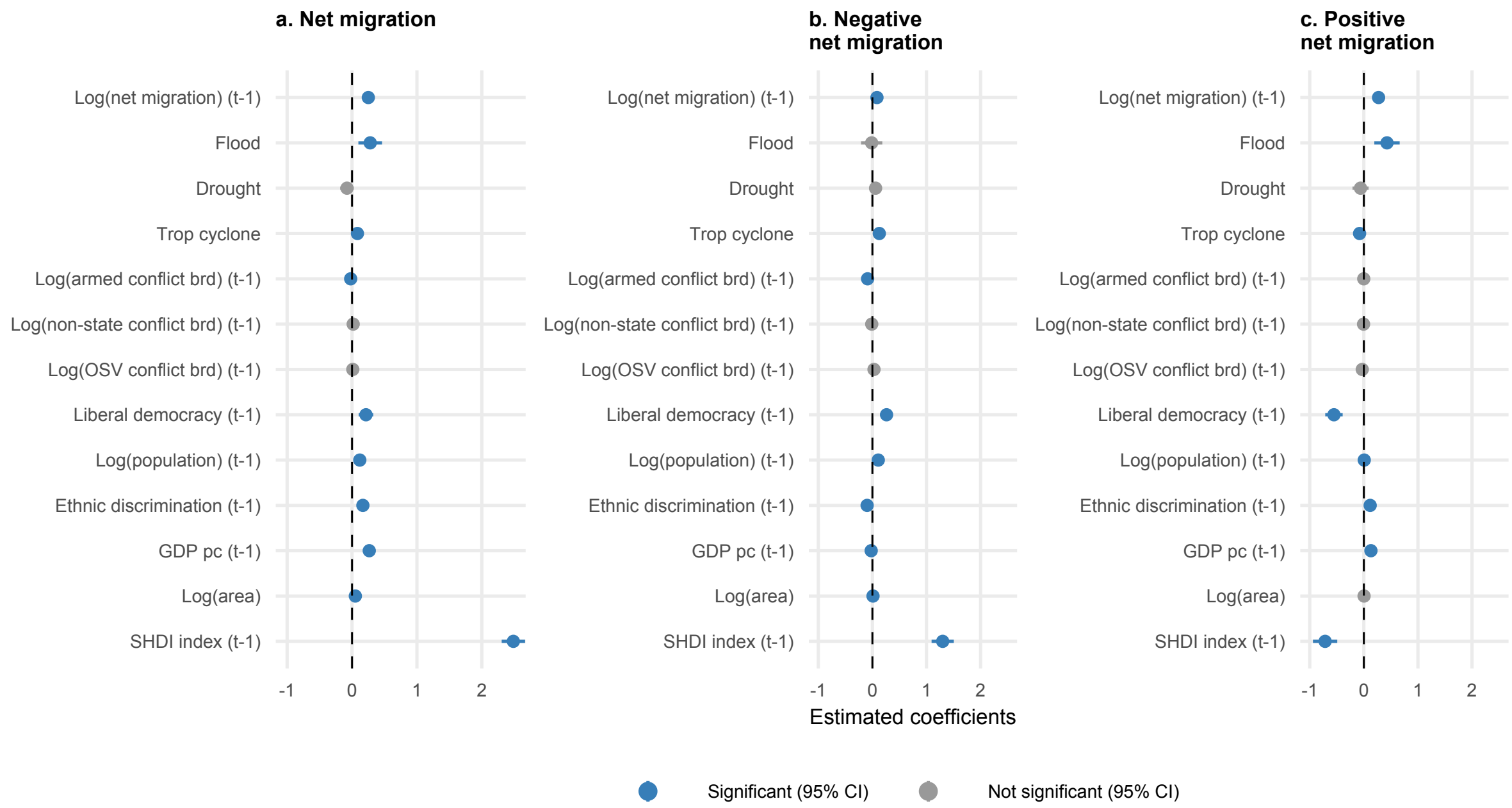

Figure S.7: Global Analysis of Single Climate Events and Net Migration. Panels present the associations between single climate extremes and net migration for (a) the full sample (n = 528,250), (b) districts with negative net migration (n = 189,887), and (c) districts with positive net migration (n = 111,467). Results show estimated coefficients from fixed-effects linear regressions with year and country fixed effects. Statistically insignificant coefficients are greyed out.

2.3 Crop failure results

Table S.4: Global Analysis of Single Climate Events and Net Migration. Results show the effect of climate extremes on net migration, including crop failure, for the full sample, negative net migration, and positive net migration, displaying coefficients for mixed effects linear regression with random intercepts for year and country.

	Dependent variable:		
	Net Migration	Negative Net Migration	Positive Net Migration
	(1)	(2)	(3)
log(net migration) ($t-1$)	0.220***	0.063***	0.225***
	(0.001)	(0.002)	(0.003)
Flood	0.368***	−0.026	0.450***
	(0.110)	(0.121)	(0.137)
Drought	−0.029	0.121*	−0.059
	(0.060)	(0.065)	(0.086)
Trop cyclone	0.075***	0.113***	−0.096***
	(0.027)	(0.032)	(0.030)
Crop failure	−0.019	0.011	−0.041
	(0.023)	(0.025)	(0.027)
log(armed conflict brd) ($t-1$)	−0.029***	−0.097***	−0.009
	(0.011)	(0.012)	(0.012)
log(non−state conflict brd) ($t-1$)	0.003	−0.032	−0.003
	(0.024)	(0.025)	(0.026)
log(OSV conflict brd) ($t-1$)	0.018	0.025	−0.015
	(0.019)	(0.021)	(0.021)
Liberal democracy ($t-1$)	0.278***	0.190***	−0.687***
	(0.073)	(0.069)	(0.092)
log(population) ($t-1$)	0.107***	0.104***	0.008**
	(0.003)	(0.004)	(0.004)
Ethnic discrimination ($t-1$)	0.199***	−0.102**	0.130**
	(0.050)	(0.052)	(0.064)
GDP pc ($t-1$)	0.279***	−0.021**	0.128***
	(0.009)	(0.010)	(0.010)
log(area)	0.052***	0.004	0.005
	(0.003)	(0.004)	(0.004)
SHDI index ($t-1$)	2.693***	1.458***	−0.835***
	(0.102)	(0.113)	(0.118)
Observations	444,766	159,890	93,860
Akaike Inf. Crit.	2,031,661.000	585,225.600	318,032.300
Bayesian Inf. Crit.	2,031,859.000	585,405.300	318,202.400
Note:			*p<0.1; **p<0.05; ***p<0.01

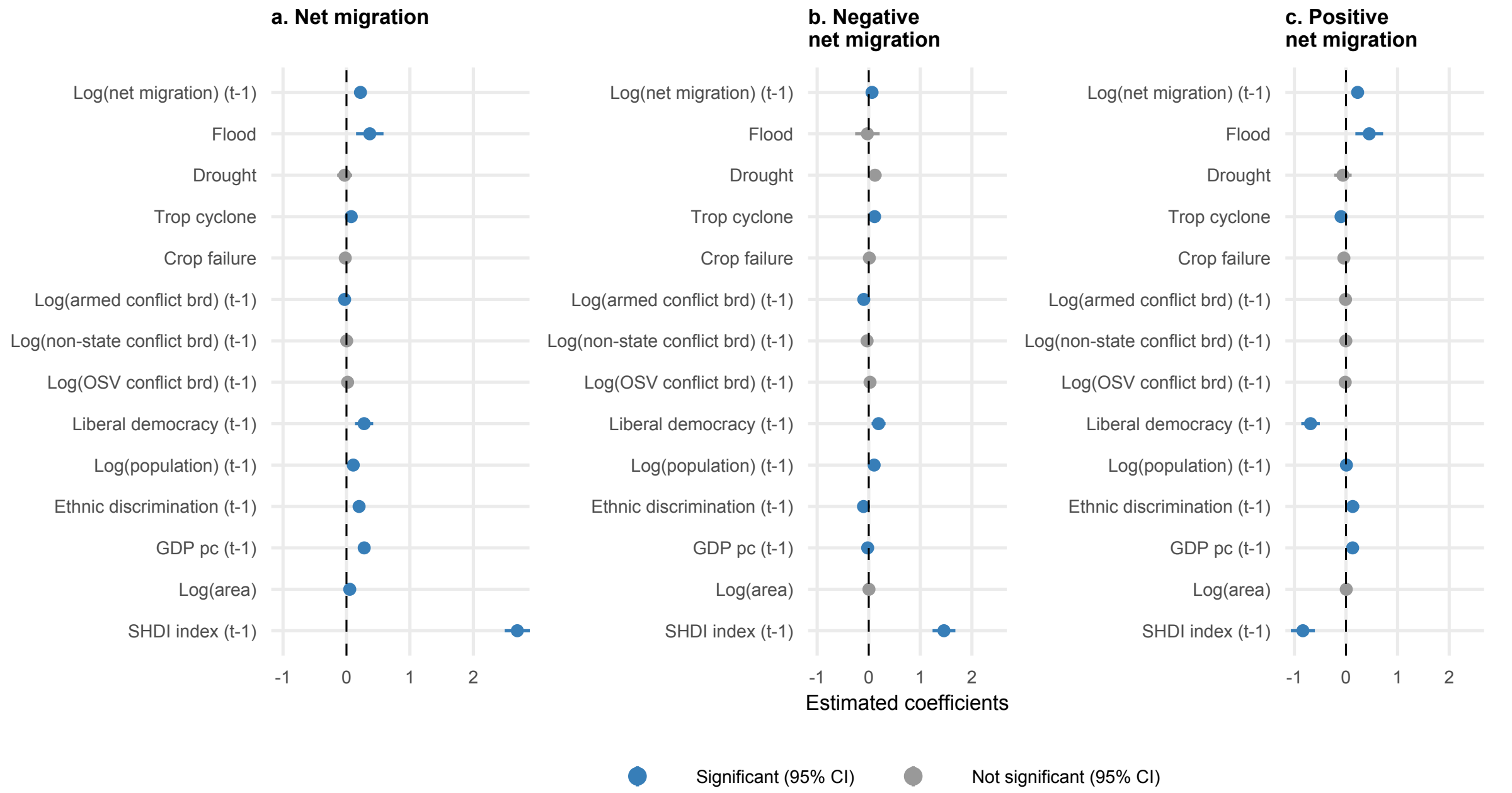

Figure S.8: Global Analysis of Single Climate Events and Net Migration. Panels present the associations between single climate extremes (including crop failure) and net migration for (a) the full sample ($n =$ 444,942), (b) districts with negative net migration ($n =$ 159,938), and (c) districts with positive net migration ($n =$ 93,908). Results show estimated coefficients from mixed-effects linear regressions with random intercepts for year and country. Statistically insignificant coefficients are greyed out.

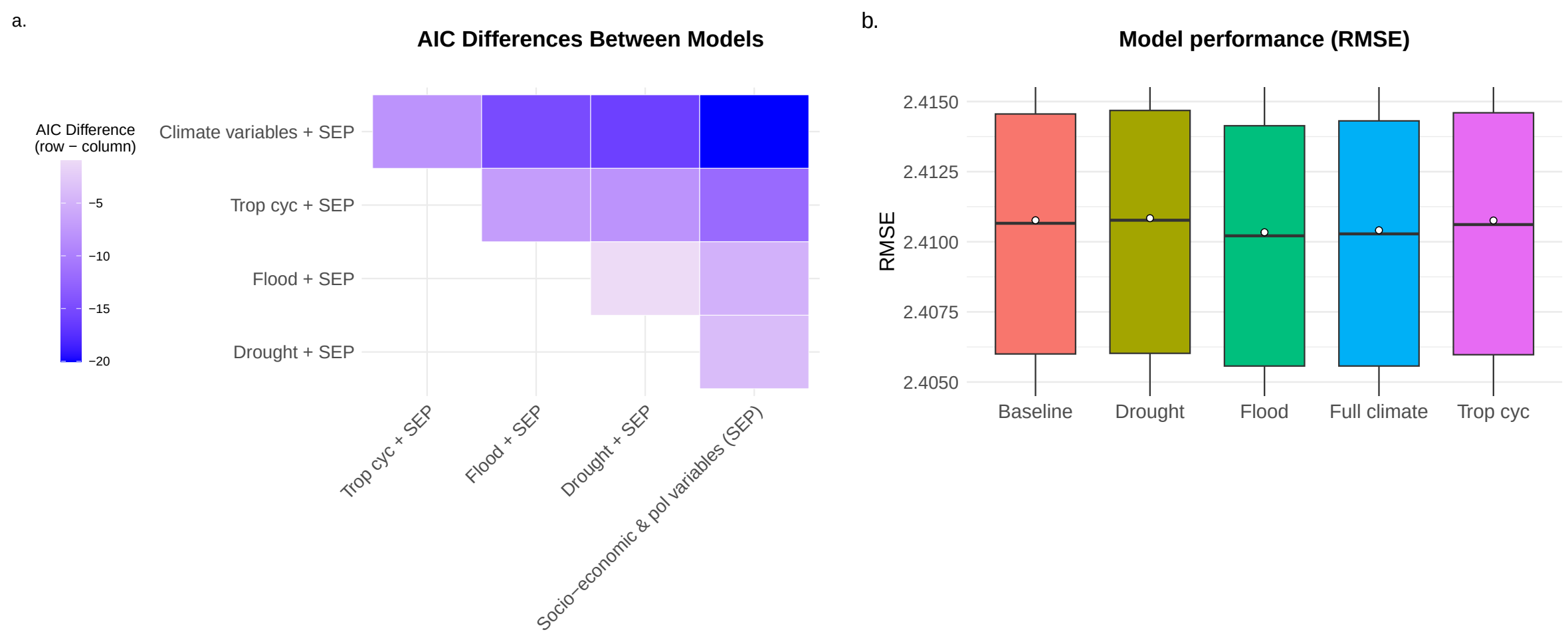

Figure S.9: a) Performance Comparison of different model specifications based on AIC, with their rankings displayed from top to bottom on the y-axis. Models are evaluated by comparing their differences in AIC, with the smallest difference (indicating a better fit) to the largest difference (indicating a poorer fit) displayed from left to right. b) Root mean square error (RMSE) comparison of different models: the boxplot presents the RMSE values obtained from 100-fold cross-validation for 5 models predicting net migration. The models include Baseline-, Drought-, Flood-, Tropical Cyclones-, and Full Climate specifications. Each boxplot displays the distribution of RMSE values for each model, with the box representing the interquartile range and the median marked inside. Error bars have been added to show the mean and associated confidence intervals

2.4 Compounded climate impact events

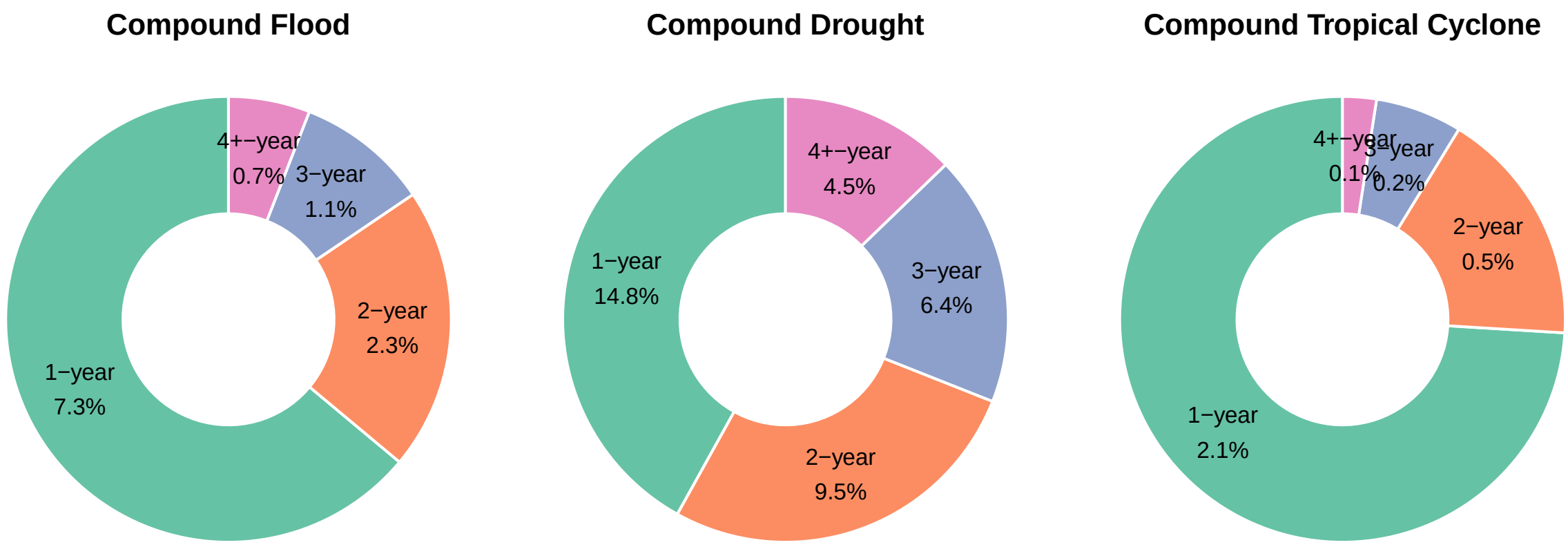

Figure S.10: Percentage of compound climate impact events over time for each event type. The percentages represent the proportion of compound climate impact events of a given duration (1-year, 2-year, 3-year, and 4-years) relative to all recorded events in the dataset, shown separately for floods, droughts, and tropical cyclones.

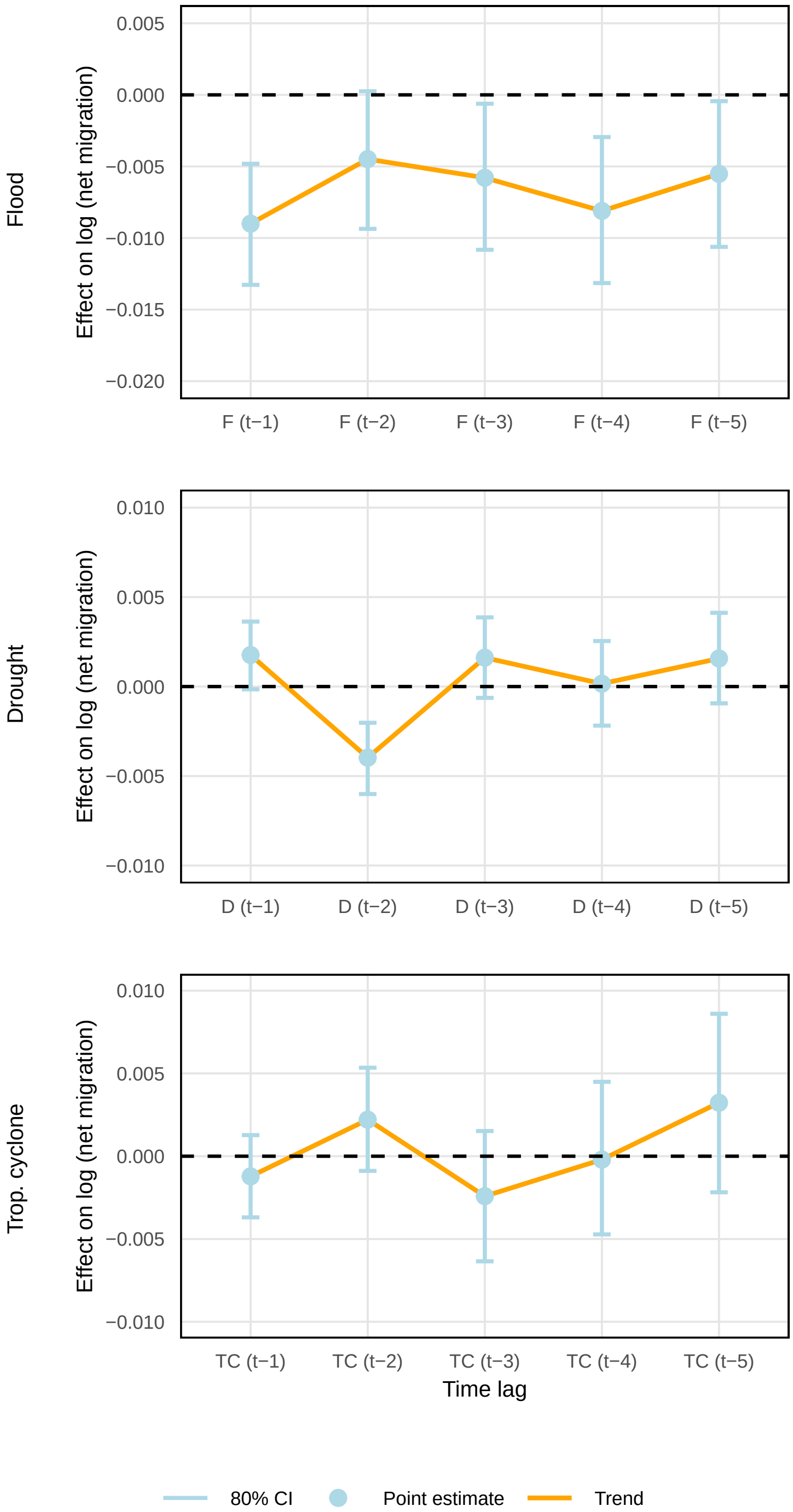

Figure S.11: Single climate events and net migration. Results show the lagged associations between single climate extremes and net migration for the full sample (n = 528,459 district-year observations), based on a hierarchical Bayesian panel vector autoregressive model (250 000 burn-in iterations, 250 000 post-burn-in iterations with thinning of 20). Points show posterior mean coefficients, and error bars represent 80% credible intervals.

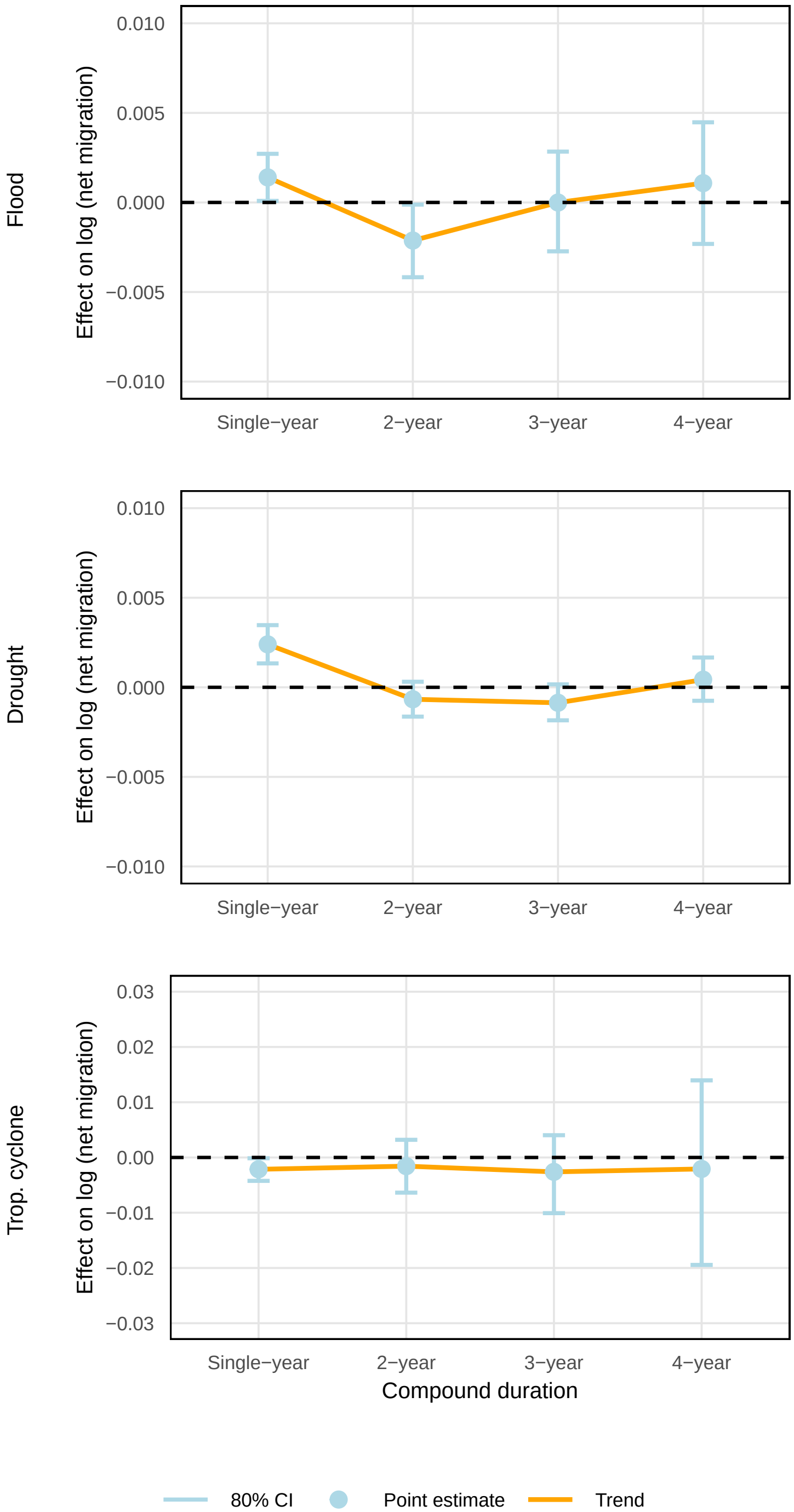

Figure S.12: Compound climate events and net migration. Results show the associations between repeated occurrences of the same type of climate extreme and net migration for the full sample ($n = 528{,}459$ district-year observations), displaying coefficients from the hierarchical Bayesian panel vector autoregressive model (12,500 burn-in iterations, 12,500 post-burn-in iterations with thinning of 20). Error bars represent 80% credible intervals.
