# Supplementary material for: Compound and heterogeneous relationships between climate extremes and global net migration
Source: Nat Clim Chang. 2026 Sep 11;16(10):1162–9. doi: 10.1038/s41558-026-02752-4 (PMC13638451; doi:10.1038/s41558-026-02752-4)
Supplement: Supplementary file 2 [file 41558_2026_2752_MOESM2_ESM.pdf]

nature portfolio

Corresponding author(s): Kristina Petrova

Last updated by author(s): 04/28/2026

# Reporting Summary

Nature Portfolio wishes to improve the reproducibility of the work that we publish. This form provides structure for consistency and transparency in reporting. For further information on Nature Portfolio policies, see our Editorial Policies and the Editorial Policy Checklist.

## Statistics

For all statistical analyses, confirm that the following items are present in the figure legend, table legend, main text, or Methods section.

| n/a | Confirmed | |
|---|---|---|
| ☐ | ☒ | The exact sample size (*n*) for each experimental group/condition, given as a discrete number and unit of measurement |
| ☐ | ☒ | A statement on whether measurements were taken from distinct samples or whether the same sample was measured repeatedly |
| ☒ | ☐ | The statistical test(s) used AND whether they are one- or two-sided<br>*Only common tests should be described solely by name; describe more complex techniques in the Methods section.* |
| ☐ | ☒ | A description of all covariates tested |
| ☐ | ☒ | A description of any assumptions or corrections, such as tests of normality and adjustment for multiple comparisons |
| ☐ | ☒ | A full description of the statistical parameters including central tendency (e.g. means) or other basic estimates (e.g. regression coefficient) AND variation (e.g. standard deviation) or associated estimates of uncertainty (e.g. confidence intervals) |
| ☐ | ☒ | For null hypothesis testing, the test statistic (e.g. *F*, *t*, *r*) with confidence intervals, effect sizes, degrees of freedom and *P* value noted<br>*Give P values as exact values whenever suitable.* |
| ☐ | ☒ | For Bayesian analysis, information on the choice of priors and Markov chain Monte Carlo settings |
| ☐ | ☒ | For hierarchical and complex designs, identification of the appropriate level for tests and full reporting of outcomes |
| ☒ | ☐ | Estimates of effect sizes (e.g. Cohen's *d*, Pearson's *r*), indicating how they were calculated |

*Our web collection on statistics for biologists contains articles on many of the points above.*

## Software and code

Policy information about availability of computer code

| | |
|---|---|
| Data collection | No primary data were collected for this study. All datasets were obtained from publicly available repositories, including ISIMIP3a, GADM, UCDP-GED, EPR (ETH Zurich), GPWv4 (SEDAC), and the Subnational Human Development Database (Scientific Data). Data extraction, cleaning, and preparation were conducted using R (version 4.4.3). |
| Data analysis | Data analysis was conducted in R (version 4.4.3). Statistical modelling and visualisation relied on standard R packages, including: BVAR, arm, brms, caret, car, data.table, dplyr, foreign, ggplot2, lme4, LRTesteR, mvtnorm, optimx, readr, reshape2, rgdal, sandwich, sf, stargazer, tidyr and tidyverse. |

For manuscripts utilizing custom algorithms or software that are central to the research but not yet described in published literature, software must be made available to editors and reviewers. We strongly encourage code deposition in a community repository (e.g. GitHub). See the Nature Portfolio guidelines for submitting code & software for further information.

## Data

Policy information about availability of data

All manuscripts must include a data availability statement. This statement should provide the following information, where applicable:

- Accession codes, unique identifiers, or web links for publicly available datasets
- A description of any restrictions on data availability
- For clinical datasets or third party data, please ensure that the statement adheres to our policy

All replication materials, including the processed datasets required to reproduce the analyses and figures in the study, are publicly available via GitHub at: https://github.com/kpppetr/district-migration-climate-impacts. All underlying raw data originate from publicly accessible third-party repositories (ISIMIP, GADM, UCDP, GPWv4, SHDI, V-Dem, etc.), each cited in the manuscript. Access to these third-party datasets is unrestricted, and no confidential data were used. The study does not involve clinical datasets or any data requiring additional access approval.

## Human research participants

Policy information about studies involving human research participants and Sex and Gender in Research.

| | |
|---|---|
| Reporting on sex and gender | Our analysis does not cover reporting on sex and gender. |
| Population characteristics | See above. |
| Recruitment | See above. |
| Ethics oversight | See above. |

Note that full information on the approval of the study protocol must also be provided in the manuscript.

# Field-specific reporting

Please select the one below that is the best fit for your research. If you are not sure, read the appropriate sections before making your selection.

☐ Life sciences ☒ Behavioural & social sciences ☐ Ecological, evolutionary & environmental sciences

For a reference copy of the document with all sections, see nature.com/documents/nr-reporting-summary-flat.pdf

# Behavioural & social sciences study design

All studies must disclose on these points even when the disclosure is negative.

| | |
|---|---|
| Study description | In our study, we rely on a quantitative research design. |
| Research sample | The study uses a quantitative global panel dataset of second-order administrative units, observed annually from 2000–2019. The sample consists of district-level observations rather than human subjects; therefore, no demographic characteristics apply. All data are derived from publicly available geospatial and socioeconomic datasets. The dataset is globally representative at the administrative-unit level. The sample is defined by the availability of harmonised net migration and climate-impact data needed to evaluate climate-migration relationships. |
| Sampling strategy | No additional sampling strategy was implemented. |
| Data collection | No primary data were collected by the authors. All datasets used in this study are secondary and publicly available datasets. |
| Timing | See above. |
| Data exclusions | See above. |
| Non-participation | See above. |
| Randomization | Not applicable. |

# Reporting for specific materials, systems and methods

We require information from authors about some types of materials, experimental systems and methods used in many studies. Here, indicate whether each material, system or method listed is relevant to your study. If you are not sure if a list item applies to your research, read the appropriate section before selecting a response.

## Materials & experimental systems

| n/a | Involved in the study |
| --- | --- |
| ☒ | ☐ Antibodies |
| ☒ | ☐ Eukaryotic cell lines |
| ☒ | ☐ Palaeontology and archaeology |
| ☒ | ☐ Animals and other organisms |
| ☒ | ☐ Clinical data |
| ☒ | ☐ Dual use research of concern |

## Methods

| n/a | Involved in the study |
| --- | --- |
| ☒ | ☐ ChIP-seq |
| ☒ | ☐ Flow cytometry |
| ☒ | ☐ MRI-based neuroimaging |
